# Founder events, isolation, and inbreeding: Intercontinental genetic structure of the domestic ferret

**DOI:** 10.1111/eva.12565

**Published:** 2017-12-20

**Authors:** Kyle D. Gustafson, Michelle G. Hawkins, Tracy L. Drazenovich, Robert Church, Susan A. Brown, Holly B. Ernest

**Affiliations:** ^1^ Wildlife Genomics and Disease Ecology Laboratory Veterinary Sciences University of Wyoming Laramie WY USA; ^2^ Department of Medicine and Epidemiology School of Veterinary Medicine, University of California–Davis Davis CA USA; ^3^ BCPhoto Columbia MO USA; ^4^ Rosehaven Exotic Animal Veterinary Services Batavia IL USA

**Keywords:** artificial selection, Australia, Europe, *Mustela putorius*, *Mustela putorius furo*, New Zealand, North America

## Abstract

Domestication and breeding for human‐desired morphological traits can reduce population genetic diversity via founder events and artificial selection, resulting in inbreeding depression and genetic disorders. The ferret (*Mustela putorius furo*) was domesticated from European polecats (*M. putorius*), transported to multiple continents, and has been artificially selected for several traits. The ferret is now a common pet, a laboratory model organism, and feral ferrets can impact native biodiversity. We hypothesized global ferret trade resulted in distinct international genetic clusters and that ferrets transported to other continents would have lower genetic diversity than ferrets from Europe because of extreme founder events and no hybridization with wild polecats or genetically diverse ferrets. To assess these hypotheses, we genotyped 765 ferrets at 31 microsatellites from 11 countries among the continents of North America, Europe, and Australia and estimated population structure and genetic diversity. Fifteen *M. putorius* were genotyped for comparison. Our study indicated ferrets exhibit geographically distinct clusters and highlights the low genetic variation in certain countries. Australian and North American clusters have the lowest genetic diversities and highest inbreeding metrics whereas the United Kingdom (UK) cluster exhibited intermediate genetic diversity. Non‐UK European ferrets had high genetic diversity, possibly a result of introgression with wild polecats. Notably, Hungarian ferrets had the highest genetic diversity and Hungary is the only country sampled with two wild polecat species. Our research has broad social, economic, and biomedical importance. Ferret owners and veterinarians should be made aware of potential inbreeding depression. Breeders in North America and Australia would benefit by incorporating genetically diverse ferrets from mainland Europe. Laboratories using ferrets as biomedical organisms should consider diversifying their genetic stock and incorporating genetic information into bioassays. These results also have forensic applications for conserving the genetics of wild polecat species and for identifying and managing sources of feral ferrets causing ecosystem damage.

## 1 INTRODUCTION

Domestication can result in a founder effect where only a few wild animals contribute to the gene pool and evolutionary trajectory of the domesticated lineage (Diamond, 2002; Mimura et al., 2017; Petersson, Jaurvi, Steffner, & Ragnarsson, 1996; Teletchea & Fontaine, 2014). Additionally, artificial selection for desirable morphological traits can further reduce genetic variation (Driscoll, Macdonald, & O'Brien, 2009; Leroy, 2011; Muñoz‐Fuentes et al., 2014). This loss of genetic diversity can result in inbreeding depression (Charlesworth & Charlesworth, 1999; Hedrick & Garcia‐Dorado, 2016; Leroy, 2014), which can be particularly problematic when a domesticated species is economically or ecologically important (González‐Recio, López de Maturana, & Gutiérrez, 2007; O'Neill, Church, McGreevy, Thomson, & Brodbelt, 2013; Rhymer & Simberloff, 1996). Thus, understanding the population structure and genetic diversity of a domestic species is critically important for long‐term persistence and can have global or regional implications based on the animal's role in society and its genetic status.

The ferret (*Mustela putorius furo* Linnaeus, 1758) was domesticated from the European polecat (*M. putorius* Linnaeus, 1758) primarily for hunting rabbits and rats (Blandford, 1987; Hosoda et al., 2000; Kurose, Abramov, & Masuda, 2000, 2008). More recently, the ferret has become a common household pet (Hernádi, Kis, Turcsán, & Topál, 2012) and, in some countries, is a laboratory model organism (Ball, 2006) or considered invasive (O'Donnell, Weston, & Monks, 2017). Selection for specific ferret coat colors has been associated with genetically determined physical abnormalities (Blaszczyk et al., 2007; Piazza, Abitbol, Gnirs, Huynh, & Cauzinille, 2014), and there is rising concern about the potential role of inbreeding and low genetic diversity in the increasing incidence of ferret cancers (Avallone et al., 2016; Bielinska, Parviainen, Kiiveri, Heikinheimo, & Wilson, 2009; Clagett, Johnston, & Han, 2016; Lewington, 2007b). Thus, for ferrets, an assessment of population structure and regional genetic diversity will be of broad social, economic, and biomedical importance.

On a global scale, pet owners and veterinarians would be informed of genetic diversity and potential inbreeding depression (Fox & Marini, 2014) and breeders trying to avoid genetic disorders would benefit by applying information about genetic diversity to regional and international breeding programs (Howard, Lynch, Santymire, Marinari, & Wildt, 2015; Willoughby et al., 2015). Biomedical laboratories using ferrets as model organisms would benefit by incorporating genetic diversity information into their bioassays because inbred individuals can be more susceptible to disease exposures (Ball, 2006; Ilmonen et al., 2008). These data could also be used to identify and conserve wild polecat species which could face reductions in genetic diversity when hybridized with feral ferrets (Costa et al., 2013; Rhymer & Simberloff, 1996). Similarly, the conservation genetics approach used here could have forensic and wildlife management applications for identifying and managing sources of feral ferrets causing ecosystem damage (O'Donnell et al., 2017; Wells, 2009).

Despite the worldwide distribution of ferrets, global patterns of domestic ferret population structure and genetic diversity have not been characterized (Costa et al., 2013; Ernest, Drazenovich, Dalbeck, & Hawkins, 2012; Thomson, 1951). Intercontinental translocations from Europe have not been well recorded, and the current understanding of ferret transportation and domestication relies heavily on transgenerational word of mouth (Church, 2007; Lewington, 2007a) and historic letters (Buller, 1877). Patterns of domestication are also clouded by the historic backcrossing with *M. putorius* (Costa et al., 2013; Davison et al., 1999; Marmi, López‐Giráldez, & Domingo‐Roura, 2004; Pitt, 1921; Poole, 1972) and potential hybridization with other polecat species (Lodé, Guiral, & Peltier, 2005; Williams et al., 1996). Although historical artificial selection for hunting ability (Carnegie, 2013; Owen, 1984) and contemporary artificial selection for coat colors and patterns (Blaszczyk et al., 2007; Lewington, 2007b; Piazza et al., 2014) likely had a large impact on ferret translocations and genetic diversity, international differences in trade laws and breeding programs could also have limited or reduced the genetic diversity of certain populations (Lee, 2002; Northern Territory Government, 2016; Queensland Government, 2016; Willoughby et al., 2015).

Our goal was to evaluate the genetic structure and levels of genetic diversity and inbreeding in pet ferrets from multiple countries among the continents of North America, Europe, and Australia. We hypothesized global ferret trade resulted in distinct international genetic clusters. We also hypothesized ferrets transported to other continents would have lower genetic diversity than ferrets from Europe because of extreme founder events and no opportunities to hybridize with wild polecats or genetically diverse ferrets. Our international and intercontinental assessment of ferret population genetics provides broad insights into global patterns of founder events, and the impacts of isolation and inbreeding on the population structure of a domesticated species (Larson & Burger, 2013). Our results will be of broad social, economic, and biomedical importance and have direct applications to the ferret industry.

## 2 MATERIALS AND METHODS

### 2.1 Sample collection and DNA extraction

Cells for DNA extraction were collected from 765 domestic ferrets (*M. putorius furo*; Australia: 222; Canada: 56; Denmark: 60; England: 63; Hungary: 19; the Netherlands: 48; Norway: 41; New Zealand: 74; Scotland: 16; Sweden: 27; United States: 139) and 15 European polecats (*M. putorius*; all from England) via buccal swab and/or hair (>10 hairs per ferret) plucked from the base of the tail (Table S1). Samples were collected by coauthors or via collaborating veterinarians between 2008 and 2011 from personal homes, rescue shelters, or breeders. During that time, cells from a single specimen that died in 2007 were collected from an Australian museum. The polecats were sampled from private breeders who had captured the individuals as wild polecats. Most polecats originated near the western border of England, which is an area known to have polecats with a high amount of introgression with ferrets (Costa et al., 2013). Any individuals known (via breeding programs) or suspected by the owners to be hybrids were removed from analyses and were not included in this sample. Swab samples were stored at −70°C, whereas hair was stored in paper envelopes at stable room temperature. DNA was extracted from buccal swabs or hair follicles from 2010 to 2011 following the exact protocols of Ernest et al. (2012).

### 2.2 PCR

Forward primers for 31 microsatellite loci (Table S2; Dallas & Piertney, 1998; Domingo‐Roura et al., 2003; Ernest et al., 2012; Lam, Gagne, & Ernest, 2016; O'Connell, Wright, & Farid, 1996; Paetkau & Strobeck, 1994) were fluorescently labeled (NED, PET, FAM, or VIC; Applied Biosystems Inc., Foster City, CA, USA). Two loci were amplified singly, and the other 29 loci were split among seven multiplexes based on fragment size and fluorescent label compatibility (Table S2). Amplifications were carried out in Bio‐Rad MyCyclers (Bio‐Rad, Hercules, CA, USA) using Ernest et al. (2012) multiplex PCR protocols (Table S2). PCR products were analyzed on an ABI 3730 capillary DNA Analyzer (Life Technologies, Carlsbad, CA, USA). Negative controls and positive controls were included with each PCR run. Fragments were visualized with STRand version 2.3.69 (Toonen & Hughes, 2001). Heterozygous and homozygous loci were run at least twice or three times, respectively.

### 2.3 Population genetic structure

We used three approaches to assess population structure, including F statistics, Bayesian population assignment models, and a discriminant analysis of principal components (DAPC). Population divergence (*F*_ST_) was calculated in GenAlEx 6.502 (Peakall & Smouse, 2006, 2012), and significance testing was based on 999 permutations. We used spatially explicit hierarchical Bayesian clustering programs GENELAND 4.0 (Guillot, Mortier, & Estoup, 2005) and TESS 2.3 (Durand, Chen, & François, 2009) to assess population assignments, and adegenet 2.0.1 (Jombart, 2008) for the DAPC.

In GENELAND, the number of populations (*K*) is a parameter optimized by the model. We followed developer recommendations for determining *K* and individual population assignments (Guillot, Estoup, Mortier, & Cosson, 2005). First, we ran 15 spatial models allowing *K* to vary from 1 to 10. All models converged on the same *K*. Thus, we ran five additional models fixing *K* at the mode and selected the model with the highest log‐likelihood to run an admixture model. Each run included 100,000 iterations, a thinning interval of 1,000, and a 25% burn‐in period prior to extracting model output.

In TESS, *K* must be specified and tested over a range of possible values. Model selection must be used to determine the *K* with the best fit to the data. We followed developer instructions for determining *K* and population assignments. First, we ran 10 nonadmixture models for each *K* from 2 to 10. For model comparisons, TESS computes a deviance information criterion (DIC). We ran 10 spatially conditional autoregressive admixture models for each *K* to the DIC plateau of nonadmixture models. All models included pairwise great circle geographic distances for weighting the Voronoi neighborhood, 100,000 iterations, and a 25% burn‐in period. We retained 20% of the models which contained the lowest DIC scores and used CLUMPP 1.1.2 to perform model averaging (Jakobsson & Rosenberg, 2007).

To assess whether spatial priors or sample size were driving population assignments (Puechmaille, 2016), we also used program STRUCTURE 2.3.4 (Pritchard et al., 2000). We randomly subsetted the samples from each country to 15, which was the number of wild polecats sampled. Using 180 subsetted samples, we ran 10 admixture models for each *K* from 1 to 10 with uniform, nonspatial priors, including 100,000 iterations and a 25% burn‐in period.

Because the algorithm for individual assignments in adegenet is not as powerful as Bayesian population assignment algorithms (Jombart, Devillard, & Balloux, 2010), we defined populations in the DAPC using countries as focal groups and treated wild European polecats as a separate group. We retained all principal components for discriminant analyses.

### 2.4 Microsatellite loci and genetic diversity

Tests for linkage disequilibrium, deviations from Hardy–Weinberg proportions, and null alleles were assessed in GENEPOP 4.5.1 (Rousset, 2008). Given the major differences in sample sizes among countries, we used genetic diversity estimates that are robust to sample size or accounted for sample size. We calculated unbiased expected heterozygosity in GenAlEx (Nei, 1978). To measure the number of alleles, we calculated allelic richness using sample size‐correcting rarefaction methods in FSTAT 2.9.3.2 (Goudet, 1995; Kalinowski, 2004). To assess inbreeding, we calculated internal relatedness using package Rhh 1.0.2 in Program R 3.3.0 (Alho, Valimaki, & Merila, 2010). Internal relatedness measures a relative outbred–inbred continuum, where negative values are suggestive of outbred individuals and positive scores are suggestive of inbreeding (Amos et al., 2001). Internal relatedness is an extension of standardized heterozygosity which standardizes estimates based on allele frequencies of the entire global sample of ferrets (Amos et al., 2001). We reported both the raw number or private alleles and the percent of private alleles, which was standardized to sample size.

## 3 RESULTS

### 3.1 Population genetic structure

All 31 loci were polymorphic among pooled samples. Only 3% of individuals had any missing data. Most individuals with missing data were missing allelic information at a single locus; however, two individuals had two missing loci, and a single individual had four missing loci. There was no evidence for null alleles or deviations from Hardy–Weinberg proportions in each assigned population after Bonferroni corrections. European polecats had the lowest *F*_ST_ values with ferrets from England and Scotland and the highest differentiation with ferrets from Australia, Canada, and the United States (Table 1). Australia had high pairwise *F*_ST_ values with every country except New Zealand and the Netherlands. European countries were not strongly differentiated but exhibited moderate differentiation with the United States and Canada. When population differentiation was assessed based on population assignments in GENELAND, the United Kingdom (UK: England & Scotland ferrets) cluster was not strongly differentiated from non‐UK European cluster (Table 1). The North American cluster was strongly differentiated from the Australian cluster and moderately differentiated from both European clusters.

**Table 1:** Summary of country‐level pairwise *F*_ST_ (below axis) and GENELAND genetic cluster‐level pairwise *F*_ST_ (above axis) estimates

Country											Genetic cluster
Australia	–							Non‐UK Europe	Australia	UK & NZ	
Canada	0.09	–						0.08	0.14	0.08	North America
Denmark	0.08	0.04	–					–	0.11	0.03	Non‐UK Europe
England	0.08	0.05	0.03	–					–	0.11	Australia
Hungary	0.07	0.06	0.03	0.05	–						
The Netherlands	0.06	0.06	0.03	0.04	0.03	–					
Norway	0.07	0.05	0.01	0.02	0.03	0.03	–				
NZ	0.06	0.07	0.04	0.03	0.05	0.05	0.02	–			
Scotland	0.09	0.07	0.03	0.01	0.05	0.04	0.02	0.04	–		
Sweden	0.09	0.05	0.02	0.04	0.06	0.05	0.02	0.05	0.05	–	
USA	0.09	0.03	0.06	0.05	0.06	0.05	0.06	0.07	0.07	0.06	–
Polecat	0.13	0.09	0.06	0.05	0.06	0.07	0.06	0.08	0.05	0.07	0.09
	Australia	Canada	Denmark	England	Hungary	The Netherlands	Norway	NZ	Scotland	Sweden	USA

UK, United Kingdom; NZ, New Zealand; USA, United States of America.

All pairwise *F*_ST_ estimates were significant (*p* < .05 based on 1,000 permutation tests) except the England–Scotland comparison.

**Figure 1:**
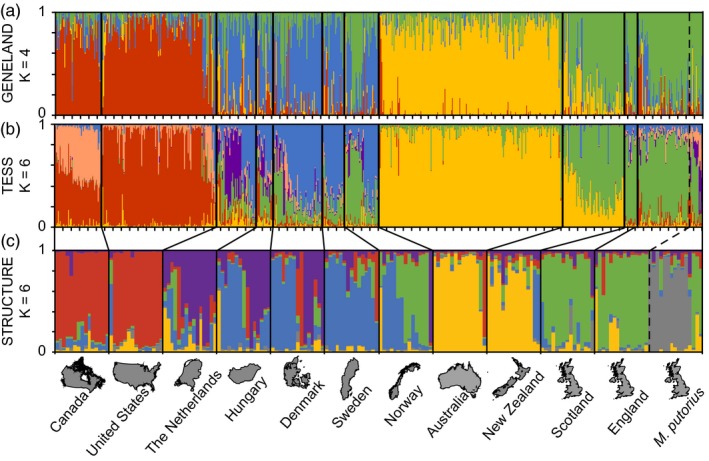
Population assignments (a–c) of ferrets (*N* = 765) and European polecats (*Mustela putorius*;* N* = 15). Program GENELAND (a) identified four genetic clusters whereas programs TESS (b) and STRUCTURE (c) identified six clusters. TESS identified additional substructure in Canada and the Netherlands. On a subsetted dataset with equal sample sizes for each country (*N* = 15), STRUCTURE assigned most of the European polecats to their own cluster. Polecats were sampled from the United Kingdom and are presented on the far right

Program GENELAND identified four genetic clusters (Figure 1a), including distinct North American and Australian clusters. Europe had two clusters, separating the UK ferrets from non‐UK European ferrets. New Zealand ferrets primarily assigned to the UK cluster, but also exhibited admixture with the Australian cluster. Norway also had a large proportion of individuals assigned to the UK cluster. Program TESS assigned individuals similarly, but found additional substructure within Canada and within the Netherlands (Figure 1b). European polecats, sampled in England, primarily assigned to the UK cluster in both programs. On a subsetted dataset, with no spatial priors and equal sample size among countries (*N* = 15), program STRUCTURE also indicated six clusters based on model probability (LnP(D); Figure 1c). The major geographic trends were similar among all programs; however, STRUCTURE assigned the majority of polecats distinctly from ferrets (Figure 1c), indicating spatial priors in GENELAND and TESS may have overridden the genetic differences among polecats and ferrets in the UK. Alternatively, the same results might have been observed if equalization of sample sizes were performed in programs GENELAND and TESS.

**Figure 2:**
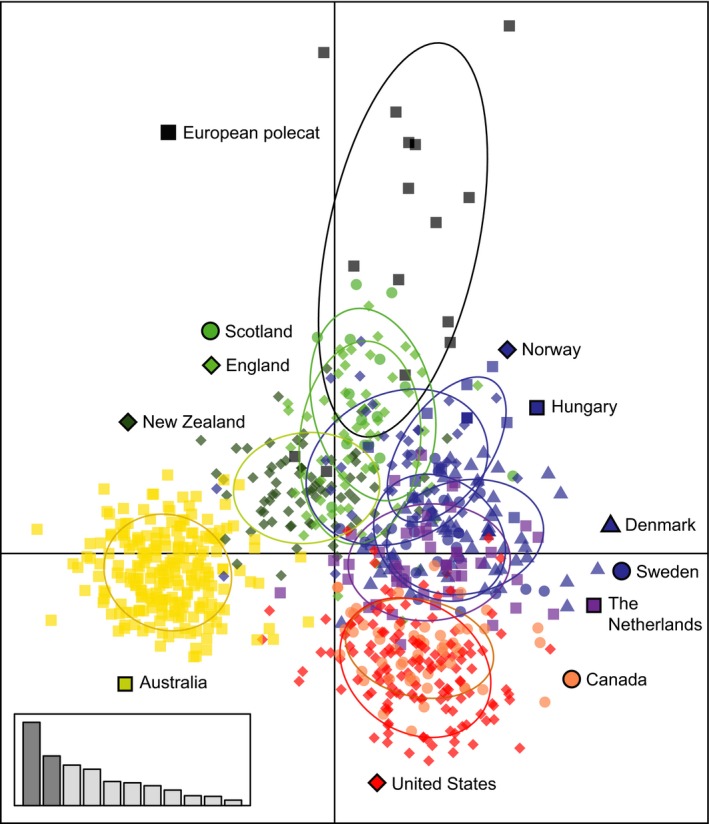
Genetic clustering of ferrets based on a discriminant analysis of principal components. Each dot is an individual ferret or polecat. Each color represents a population identified by program TESS (Figure 1b). European polecats are represented as black squares for easier visualization and based on STRUCTURE analyses (Figure 1c). Discriminant function 1 (*x*‐axis) accounted for 26.3% of the variation and discriminant function 2 (*y*‐axis) accounted for 15.7%. The inset barplot shows which axes are being displayed and the relative proportion of variation explained by each of the nine discriminant functions. Two‐thirds of the individuals in each country are contained within the corresponding ellipsoid

The DAPC showed similar patterns to the previous analyses and supports the genetic distinction between ferrets and polecats in the UK (Figure 2). Australia had the least amount of overlap with any other country, indicated by DAPC axis 1 (26.3% of total variation), which primarily separated Australia from all other countries. Polecats clustered most closely to ferrets in the UK, with some overlap. Australian and North American ferrets are most distinct from sampled European polecats, and then ferrets from the UK, indicated on DAPC axis 2 (15.7%). Similar to Bayesian analyses, New Zealand clustered between UK and Australian ferrets, but most closely to the UK. Ferrets from Hungary and Norway also clustered closely to the UK. Ferrets from Denmark, Sweden, and the Netherlands clustered together between UK and North American ferrets.

### 3.2 Microsatellite loci and genetic diversity

Based on genetic diversity estimates that are robust to sample size or account for sample size, ferrets from Australia, Canada, and the United States had the lowest genetic diversity (Table 2). Australian ferrets, despite having the largest sample size, had only a single private allele and the lowest number of polymorphic loci. North American ferrets had no private alleles and, with Australia, shared among the lowest measures of allelic richness, Shannon index of allelic diversity, and heterozygosities. Ferrets from the United States and Australia, respectively, had the highest measures of internal relatedness (i.e., inbreeding). New Zealand, England, and Scotland ferrets had intermediate levels of genetic diversity. Ferrets from all other European countries had relatively high estimates of genetic diversity and Hungarian ferrets had the highest allelic richness, number of private alleles, Shannon index, heterozygosities, and the lowest internal relatedness. Notably, ferrets from all countries had mean positive values for internal relatedness, indicative of universal inbreeding.

**Table 2:** Genetic diversity of ferrets and European polecats

Country	*N*	A_r_	*SE*	Pr_A_	Po_L_	I	*SE*	H_O_	*SE*	uH_E_	*SE*	IR	*SE*
European polecat (*Mustela putorius*)													
England	15	3.19	0.24	2	100	0.82	0.06	0.41	0.03	0.48	0.04	0.17	0.07
Domestic ferret (*M. putorius furo*)													
Australia	222	2.25	0.05	1	90	0.54	0.05	0.30	0.03	0.32	0.03	0.23	0.01
Canada	56	2.27	0.10	0	94	0.56	0.05	0.34	0.03	0.35	0.03	0.16	0.02
Denmark	60	3.15	0.11	8	100	0.81	0.05	0.39	0.03	0.47	0.02	0.15	0.03
England	63	2.90	0.10	5	97	0.69	0.05	0.34	0.03	0.39	0.03	0.20	0.03
Hungary	19	3.43	0.27	9	97	0.83	0.06	0.43	0.03	0.48	0.03	0.07	0.03
The Netherlands	48	2.97	0.12	4	100	0.78	0.05	0.38	0.03	0.46	0.03	0.16	0.03
Norway	41	3.11	0.13	1	100	0.78	0.05	0.40	0.02	0.45	0.03	0.11	0.04
New Zealand	74	2.63	0.09	3	100	0.64	0.05	0.33	0.03	0.37	0.03	0.21	0.02
Scotland	16	2.87	0.23	1	97	0.67	0.05	0.39	0.04	0.41	0.03	0.10	0.05
Sweden	27	2.83	0.15	2	100	0.74	0.05	0.41	0.04	0.45	0.03	0.10	0.04
United States	139	2.40	0.05	0	100	0.56	0.05	0.30	0.03	0.33	0.03	0.25	0.02

*N*, sample size; A_r_, sample size‐corrected allelic richness; Pr_A_, raw private alleles; Po_L_, percent of polymorphic loci standardized to sample size; I, Shannon index; H_O_, observed heterozygosity; uH_E_, unbiased expected heterozygosity which is robust to sample size differences; IR, average individual internal relatedness based on the entire sample of ferrets and polecats; *SE*, standard error.

## 4 DISCUSSION

Intercontinental ferret (*Mustela putorius furo*) trade has resulted in geographically distinct genetic clusters, likely resulting from founder events and geographic isolation combined with inbreeding and genetic drift. Spatially explicit programs TESS and GENELAND showed comparable population structure, however, TESS identified additional substructure within North America and Hungary. Additionally, our nonspatial STRUCTURE results were highly consistent with those from TESS and GENELAND except that STRUCTURE identified the sampled polecats to be genetically distinct from domestic ferrets, indicating the algorithms from the spatial programs or unequal sample sizes may have overridden the genetic differences between polecats and ferrets in the UK. Within our sample, ferrets within any single country tended to assign to only one of the four genetic clusters, including the United Kingdom (UK), non‐UK Europe, North America, or the Australia cluster. Exceptions to this pattern were New Zealand and Norway. Ferrets in New Zealand primarily assigned to the UK cluster but also shared assignments with the Australian cluster, supporting the hypothesis that New Zealand ferrets originated from England and from Australia (Buller, 1877). Ferrets from Norway either assigned to the UK or the non‐UK European cluster. The four major clusters showed extreme variation in genetic diversity and inbreeding measures. Ferrets in Europe had higher levels of genetic diversity than ferrets on other continents. Australia, Canada, and the United States had ferrets with the lowest genetic diversity and highest inbreeding measures. New Zealand, because of its shared ancestry, had low levels of genetic diversity, but greater diversity than Australian ferrets.

European polecats all exhibited variation at the microsatellite loci developed from the domestic ferret (Ernest et al., 2012), implying common ancestry (Blandford, 1987; Hosoda et al., 2000; Kurose et al., 2000, 2008). However, we did not sample other polecat species and therefore cannot determine whether the domestic ferret was domesticated from other possible species. Identifying the source location for each domestic cluster remains difficult because we do not have samples from each European country where ferrets are present and ferrets in Europe could potentially be hybridized with wild polecats or potentially other species (Costa et al., 2013; Lodé et al., 2005; Pitt, 1921; Poole, 1972), reducing any signal of relationships (Davison et al., 1999; Marmi et al., 2004). Although spatial population assignment models did not differentiate polecats from ferrets, a subsampled dataset with equal sample sizes and uniform priors indicated a genetic distinction, which is consistent with the DAPC.

A previous study observed high expected heterozygosities (mean ± *SE*: 0.58 ± 0.12) and allelic richness (3.93 ± 0.13) among populations of European polecats from mainland Europe (Pertoldi et al., 2006). Cross‐breeding between ferrets and genetically diverse polecats could explain the high genetic diversity observed in non‐UK European countries. In contrast, polecats in the UK experienced a dramatic reduction in population size during the 19th century (Langley & Yalden, 1977), leading to a genetic bottleneck (Costa et al., 2013). Our observations of genetic diversity for polecats from UK are consistent with previous reports (Costa et al., 2013), which are higher than those of domestic ferrets but lower than polecats from mainland Europe (Moller et al., 2004; Pertoldi et al., 2006). Ferret hybridization is common in the UK but does not seem to be increasing genetic diversity in pet ferrets, possibly because ferret hybridization is occurring with a genetically recovering polecat population that recently went through a bottleneck (Costa et al., 2013). Additionally, three of the 15 polecats assigned as UK ferrets using STRUCTURE, which could indicate we sampled 12 wild polecats and three feral ferrets. Combined, this could explain the high internal relatedness observed in polecats, despite the high genetic diversity. Notably, Hungarian then Danish ferrets had the most private alleles and the highest measures of genetic diversity. Hungary is the only country we sampled with both European polecats (*M. putorius*) and Steppe polecats (*M. eversmanii* Lesson, 1872) (Lanszki & Heltai, 2007; Šálek et al., 2013). Similarly, Denmark has both European polecats and American mink (*Neovision vison* Schreber, 1777) (Hammershøj et al.,^2006^). Although we do not have direct evidence for hybridization, the mechanism leading to unique alleles in these countries could be a result of hybridization or large effective population sizes.

None of the countries in which we sampled ferrets had an expected heterozygosity (mean among all domestic ferrets in our dataset: 0.41 ± 0.10) or allelic richness (mean among all domestic ferrets in our dataset: 2.80 ± 0.10) approaching those of their wild counterparts (Moller et al., 2004; Pertoldi et al., 2006), indicating founder events, genetic drift, and/or inbreeding have affected ferret genetic diversity (Diamond, 2002; Petersson et al., 1996; Teletchea & Fontaine, 2014). In natural systems, internal relatedness values range from negative (i.e., genetically outbred) to positive (i.e., genetically inbred; Amos et al., 2001). Ferrets from all countries exhibited mean positive internal relatedness, indicative of widespread inbreeding. Artificial selection is well known to reduce genetic diversity (Driscoll et al., 2009; Leroy, 2011; Muñoz‐Fuentes et al., 2014) and breeding for ferret coat color has been shown to be associated with genetically determined physical abnormalities (Blaszczyk et al., 2007; Piazza et al., 2014), which could be an indication of inbreeding depression (Charlesworth & Charlesworth, 1999; Hedrick & Garcia‐Dorado, 2016; Leroy, 2014). Cancer rates are increasing in pet ferret populations (Antinoff & Williams, 2012; Bakthavatchalu, Muthupalani, Marini, & Fox, 2016), and although the mechanism for increasing cancer rates is currently unknown (Fox, Muthupalani, Kiupel, & Williams, 2014), inbreeding and low genetic diversity are suspected (Bakthavatchalu et al., 2016; Bielinska et al., 2009). If lack of genetic diversity is a contributor to cancer acquisition, as in other systems (Epstein et al., 2016; McAloose & Newton, 2009; Morris, Wright, Grueber, Hogg, & Belov, 2015; Rahman, 2014), ferrets in Australia, Canada, New Zealand, and the United States are most at risk.

One of the primary ways to reverse inbreeding depression is through genetic restoration (Frankham, 2015; Whiteley, Fitzpatrick, Funk, & Tallmon, 2015). In domestic animal populations, genetic restoration requires human intervention and breeding programs (Ralls & Ballou, 1986; Rollinson et al., 2014). However, international trade laws could limit the feasibility of introducing new genetic material to genetically depauperate pet ferret populations, especially on continents without potential for interbreeding with wild polecats. For example, ferrets are common pets in Australia (Talbot, Freire, & Wassens, 2014) and our study supports the assertion that inbreeding has been a national problem (Lewington, 2007b). However, ferret importation is currently illegal in Australia (Department of the Environment and Energy, 2017) and ferrets are completely prohibited in Queensland (Queensland Government, 2016) and the Northern Territory (Northern Territory Government, 2016). Despite New Zealand allowing ferret exportation for permitted breeders, the purchase and importation of ferrets were banned because of damage to native species (Lee, 2002; O'Donnell et al., 2017; Wells, 2009). Thus, Australia and New Zealand breeders should actively minimize inbreeding among currently available ferrets.

We do not see any particular utility for maintaining genetically distinct ferret clusters in most cases. Thus, for countries with inbred ferrets, we recommend that pet and laboratory breeding programs should incorporate ferrets from other countries. Although outbreeding depression could result from such crosses, the potential for inbreeding depression is much more likely (Rollinson et al., 2014). Additionally, it could also be genetically beneficial for breeding programs to introduce new genes into their domestic lines through ethical and legal cross‐breeding with wild polecats. However, this could potentially result in ferrets with different behaviors than those that are considered desirable for pets (Hernádi et al., 2012) or the introduction of unwanted diseases. If this action is taken, breeders should ensure ferrets do not breed into the wild population, which could put the wild population at risk of genetic disorders (Costa et al., 2013; Rhymer & Simberloff, 1996). To reduce potential risk of inbreeding depression, ferret breeding programs designed for specific coat colors should consider mating unrelated individuals and occasionally “diluting” specific coat‐color lines with individuals of different varieties.

The United States and Canada allow ferret importation (Animal and Plant Health Inspection Service, 2016; Canadian Food Inspection Agency, 2015), yet have among the lowest measures of genetic diversity observed. Despite the large sample size, North American ferrets had no private alleles, providing no evidence of hybridization with native black‐footed ferrets (Williams et al., 1996). The opportunity for increasing domestic ferret genetic diversity in North America is currently available and should be considered given the application of ferrets as model organisms in North American biomedical laboratories (Ball, 2006; Jones et al., 2017; Peng et al., 2014; Porter, 2016; Vanchieri, 2001). Researchers should consider the effect low genetic diversity and inbreeding can have on the results and inferences from disease exposure experiments (Spielman, Brook, Briscoe, & Frankham, 2004; Whiteman, Matson, Bollmer, & Parker, 2006). Although causal links between neutral genetic markers and neoplasms cannot be made, our research highlights the lack of genetic variation present in certain ferret clusters. With the sequencing of the ferret genome being recently completed (Peng et al., 2014), researchers can now search the genome for specific genes which may be linked to cancer acquisition in this developing cancer model system (Aizawa et al., 2013, 2016).

Overall, our study identified international and intercontinental domestic ferret population structure and indicates Australian and North American ferrets have the lowest genetic diversities and are most highly diverged from the European polecat. Given current importation bans, New Zealand ferrets are essentially isolated and, in the future, may also exhibit patterns of low genetic diversity and inbreeding. Prevention or mitigation of inbreeding depression will require international cooperation among breeding programs and should include genetically diverse ferrets from mainland Europe. In some cases, political factors limit the ability of potential international breeding programs. This research highlights the need for politicians, coat‐oriented breeders, and laboratory‐stock breeders to reassess their policies. Given the close relatedness among domestic ferrets, European polecats, Steppe polecats, black‐footed ferrets, American mink, and European mink (Cabria et al., 2011; Kurose et al., 2008; Lodé et al., 2005; Williams et al., 1996), the conservation genetics approach used in this study has practical applications for conserving the genetic diversity of related wild species that could face reduction in genetic diversity when hybridized with feral ferrets (Bonesi & Palazon, 2007; Cabria et al., 2015; Šálek et al., 2013; Wisely, Buskirk, Fleming, McDonald, & Ostrander, 2002) and for the identification and management of feral ferret source populations causing ecosystem damage (Bodey, Bearhop, & McDonald, 2011; Byrom, Caley, Paterson, & Nugent, 2015; O'Donnell et al., 2017; Wells, 2009).

## DATA ARCHIVING STATEMENT

Genotype data available from the Dryad Digital Repository: https://doi.org/10.5061/dryad.24c24
