# Supplementary material for: Founder events, isolation, and inbreeding: Intercontinental genetic structure of the domestic ferret
Source: Evol Appl. 2017 Dec 20;11(5):694–704. doi: 10.1111/eva.12565 (PMC5979634; doi:10.1111/eva.12565)
Supplement: Supplementary file 1 [file EVA-11-694-s001.docx]

Founder events, isolation, and inbreeding: Intercontinental genetic structure of the domestic ferret

*Evolutionary Applications*

**Table S1** Summary of collected domestic ferrets and polecats, including the sample ID, date collected, sex, domestic or polecat, and location.

| Sample | Collected | Sex | Type | Town | State | Country |
| --- | --- | --- | --- | --- | --- | --- |
| 1 | 02/13/08 | F | domestic | Sacramento | CA | USA |
| 2 | 02/13/08 | F | domestic | Sacramento | CA | USA |
| 3 | 02/13/08 | M | domestic | Sacramento | CA | USA |
| 4 | 02/13/08 | M | domestic | Sacramento | CA | USA |
| 5 | 02/13/08 | M | domestic | Sacramento | CA | USA |
| 6 | 02/13/08 | M | domestic | Sacramento | CA | USA |
| 7 | 02/13/08 | F | domestic | Sacramento | CA | USA |
| 8 | 02/13/08 | F | domestic | Sacramento | CA | USA |
| 9 | 02/13/08 | F | domestic | Sacramento | CA | USA |
| 10 | 02/13/08 | F | domestic | Sacramento | CA | USA |
| 11 | 05/20/08 | M | domestic | Port Kennedy | Western Australia | Australia |
| 14 | 05/20/08 | ? | domestic | Stratton | Western Australia | Australia |
| 15 | 05/20/08 | F | domestic | Stratton | Western Australia | Australia |
| 17 | 05/20/08 | M | domestic | Stratton | Western Australia | Australia |
| 18 | 05/19/08 | M | domestic | Greenwood | Western Australia | Australia |
| 19 | 05/19/08 | M | domestic | Two Rocks | Western Australia | Australia |
| 21 | 05/24/08 | F | domestic | Greenwood | Western Australia | Australia |
| 22 | 05/24/08 | F | domestic | Melborne | Victoria | Australia |
| 23 | 05/07/08 | F | domestic | Sandy Bay | Tasmania | Australia |
| 24 | 05/19/08 | F | domestic | Greenwood | Western Australia | Australia |
| 25 | 05/13/08 | M | domestic | Fraser | ACT | Australia |
| 26 | 05/13/08 | F | domestic | Gordon | ACT | Australia |
| 27 | 05/13/08 | M | domestic | Gordon | ACT | Australia |
| 30 | 05/19/08 | M | domestic | Greenwood | Western Australia | Australia |
| 32 | 05/19/08 | M | domestic | Greenwood | Western Australia | Australia |
| 35 | 05/13/08 | M | domestic | Charnwood | ACT | Australia |
| 36 | 05/08/08 | M | domestic | Brisbane | Queensland | Australia |
| 37 | 05/20/08 | F | domestic | Stratton | Western Australia | Australia |
| 39 | 05/19/08 | F | domestic | Two Rocks | Western Australia | Australia |
| 40 | 05/07/08 | M | domestic | Sandy Bay | Tasmania | Australia |
| 43 | 05/20/08 | M | domestic | Stratton | Western Australia | Australia |
| 47 | 05/07/08 | F | domestic | Hobart | Tasmania | Australia |
| 48 | 05/07/08 | F | domestic | Oatlands | Tasmania | Australia |
| 52 | 05/12/08 | F | domestic | Seven Hills | New South Wales | Australia |
| 53 | 08/01/08 | M | domestic | Tingstade | Gotlands Lan | Sweden |
| 55 | 05/13/08 | M | domestic | Kambah | ACT | Australia |
| 62 | 08/01/08 | F | domestic | Copenhagen | Hovedstaden | Denmark |
| 66 | 05/20/08 | M | domestic | Forrestfield | Western Australia | Australia |
| 68 | 05/07/08 | F | domestic | Hobart | Tasmania | Australia |
| 76 | 05/21/08 | M | domestic | Hamersley | Western Australia | Australia |
| 78 | 05/13/08 | F | domestic | Kambah | ACT | Australia |
| 79 | 05/19/08 | F | domestic | Greenwood | Western Australia | Australia |
| 81 | 05/19/08 | M | domestic | Greenwood | Western Australia | Australia |
| 82 | 05/01/08 | F | domestic | Brisbane | Queensland | Australia |
| 83 | 05/17/08 | F | domestic | Narangba | Queensland | Australia |
| 84 | 05/12/08 | F | domestic | St. Mary's | New South Wales | Australia |
| 86 | 05/19/08 | F | domestic | Greenwood | Western Australia | Australia |
| 91 | 04/01/08 | F | domestic | Riparon | North Island | NZ |
| 92 | 05/07/08 | M | domestic | Mornington | Tasmania | Australia |
| 93 | 08/01/08 | M | domestic | Copenhagen | Hovedstaden | Denmark |
| 94 | 03/28/08 | F | domestic | Hamilton | North Island | NZ |
| 95 | 05/19/08 | M | domestic | Greenwood | Western Australia | Australia |
| 96 | 05/20/08 | F | domestic | Port Kennedy | Western Australia | Australia |
| 97 | 04/01/08 | F | domestic | Riparon | North Island | NZ |
| 98 | 05/20/08 | F | domestic | Stratton | Western Australia | Australia |
| 99 | 03/28/08 | M | domestic | Cambridge | North Island | NZ |
| 100 | 03/28/08 | F | domestic | Cambridge | North Island | NZ |
| 101 | 05/13/08 | F | domestic | Charnwood | ACT | Australia |
| 102 | 03/28/08 | F | domestic | Hamilton | North Island | NZ |
| 103 | 03/28/08 | M | domestic | Cambridge | North Island | NZ |
| 104 | 03/28/08 | M | domestic | Hamilton | North Island | NZ |
| 105 | 07/13/08 | M | domestic | Denton | North West England | England |
| 107 | 05/13/08 | M | domestic | Charnwood | ACT | Australia |
| 108 | 05/13/08 | M | domestic | McKellar | ACT | Australia |
| 109 | 05/19/08 | F | domestic | Greenwood | Western Australia | Australia |
| 110 | 05/20/08 | F | domestic | Forrestfield | Western Australia | Australia |
| 112 | 05/13/08 | F | domestic | Charnwood | ACT | Australia |
| 114 | 05/08/08 | M | domestic | Upper Blessington | Tasmania | Australia |
| 116 | 05/19/08 | M | domestic | Wanneroo | Western Australia | Australia |
| 117 | 05/19/08 | M | domestic | Greenwood | Western Australia | Australia |
| 120 | 05/19/08 | M | domestic | Two Rocks | Western Australia | Australia |
| 121 | 05/20/08 | M | domestic | Armdale | Western Australia | Australia |
| 122 | 08/01/08 | M | domestic | Tastrup | Hovedstaden | Denmark |
| 125 | 04/01/08 | M | domestic | Riparon | North Island | NZ |
| 127 | 05/19/08 | F | domestic | Two Rocks | Western Australia | Australia |
| 128 | 05/07/08 | M | domestic | Bagdad | Tasmania | Australia |
| 129 | 05/13/08 | M | domestic | Charnwood | ACT | Australia |
| 130 | 05/19/08 | F | domestic | Wanneroo | Western Australia | Australia |
| 131 | 05/20/08 | F | domestic | Port Kennedy | Western Australia | Australia |
| 133 | 05/20/08 | M | domestic | Forrestfield | Western Australia | Australia |
| 134 | 05/20/08 | F | domestic | Armdale | Western Australia | Australia |
| 135 | 04/01/08 | M | domestic | Riparon | North Island | NZ |
| 136 | 04/01/08 | F | domestic | Rangitaiki | North Island | NZ |
| 137 | 04/01/08 | F | domestic | Rangitaiki | North Island | NZ |
| 140 | 05/19/08 | F | domestic | Wanneroo | Western Australia | Australia |
| 141 | 05/20/08 | F | domestic | Forrestfield | Western Australia | Australia |
| 142 | 05/19/08 | M | domestic | Greenwood | Western Australia | Australia |
| 144 | 05/13/08 | F | domestic | Charnwood | ACT | Australia |
| 146 | 05/19/08 | F | domestic | Greenwood | Western Australia | Australia |
| 148 | 05/07/08 | F | domestic | Hobart | Tasmania | Australia |
| 150 | 05/19/08 | M | domestic | Greenwood | Western Australia | Australia |
| 152 | 05/13/08 | M | domestic | Charnwood | ACT | Australia |
| 153 | 05/12/08 | F | domestic | South Penrith | New South Wales | Australia |
| 155 | 05/13/08 | F | domestic | Gordon | ACT | Australia |
| 157 | 05/19/08 | M | domestic | Two Rocks | Western Australia | Australia |
| 160 | 04/01/08 | F | domestic | Rangitaiki | North Island | NZ |
| 161 | 05/13/08 | F | domestic | McKellar | ACT | Australia |
| 162 | 05/19/08 | M | domestic | Karratha | Western Australia | Australia |
| 163 | 08/01/08 | F | domestic | Valby | Hovedstaden | Denmark |
| 167 | 05/20/08 | M | domestic | Stratton | Western Australia | Australia |
| 169 | 05/01/08 | M | domestic | Brisbane | Queensland | Australia |
| 171 | 05/19/08 | F | domestic | Greenwood | Western Australia | Australia |
| 175 | 05/20/08 | F | domestic | Forrestfield | Western Australia | Australia |
| 180 | 05/19/08 | M | domestic | Greenwood | Western Australia | Australia |
| 181 | 05/13/08 | M | domestic | Kambah | ACT | Australia |
| 182 | 05/12/08 | F | domestic | South Penrith | New South Wales | Australia |
| 184 | 04/01/08 | F | domestic | Rangitaiki | North Island | NZ |
| 185 | 05/13/08 | M | domestic | Rivett | Western Australia | Australia |
| 187 | 03/28/08 | M | domestic | Cambridge | North Island | NZ |
| 188 | 05/13/08 | F | domestic | Ngunnawal | ACT | Australia |
| 189 | 05/12/08 | F | domestic | Padstow | New South Wales | Australia |
| 190 | 03/28/08 | M | domestic | Cambridge | North Island | NZ |
| 191 | 05/12/08 | F | domestic | Sydney | New South Wales | Australia |
| 193 | 03/28/08 | F | domestic | Cambridge | North Island | NZ |
| 196 | 05/13/08 | M | domestic | Torrens | ACT | Australia |
| 197 | 05/13/08 | M | domestic | Rivett | Western Australia | Australia |
| 198 | 05/13/08 | F | domestic | Ngunnawal | ACT | Australia |
| 200 | 05/13/08 | M | domestic | Queanbeyan | New South Wales | Australia |
| 201 | 05/18/08 | M | domestic | Macleay Island | Queensland | Australia |
| 205 | 05/12/08 | M | domestic | Padstow | New South Wales | Australia |
| 206 | 05/12/08 | M | domestic | Padstow | New South Wales | Australia |
| 209 | 05/07/08 | F | domestic | Hobart | Tasmania | Australia |
| 210 | 05/19/08 | M | domestic | Greenwood | Western Australia | Australia |
| 212 | 05/12/08 | M | domestic | Padstow | New South Wales | Australia |
| 213 | 05/20/08 | M | domestic | Perth | Western Australia | Australia |
| 214 | 05/12/08 | F | domestic | Springwood | New South Wales | Australia |
| 215 | 05/13/08 | F | domestic | Kambah | ACT | Australia |
| 216 | 05/13/08 | F | domestic | Fraser | ACT | Australia |
| 217 | 05/13/08 | F | domestic | Dunlop | ACT | Australia |
| 218 | 05/13/08 | F | domestic | Bomaderry | New South Wales | Australia |
| 221 | 05/12/08 | F | domestic | Sydney | New South Wales | Australia |
| 222 | 05/13/08 | M | domestic | Dunlop | ACT | Australia |
| 226 | 05/13/08 | M | domestic | Dunlop | ACT | Australia |
| 227 | 03/28/08 | M | domestic | Cambridge | North Island | NZ |
| 228 | 03/28/08 | F | domestic | Cambridge | North Island | NZ |
| 230 | 04/01/08 | M | domestic | Riparon | North Island | NZ |
| 231 | 04/01/08 | F | domestic | Rangitaiki | North Island | NZ |
| 232 | 04/01/08 | M | domestic | Rangitaiki | North Island | NZ |
| 233 | 04/01/08 | F | domestic | Rangitaiki | North Island | NZ |
| 234 | 05/01/08 | F | domestic | Brisbane | Queensland | Australia |
| 235 | 05/13/08 | M | domestic | Charnwood | ACT | Australia |
| 236 | 05/12/08 | F | domestic | Penrith | New South Wales | Australia |
| 237 | 04/01/08 | F | domestic | Rangitaiki | North Island | NZ |
| 238 | 03/28/08 | M | domestic | Auckland | North Island | NZ |
| 241 | 05/08/08 | F | domestic | Hastings | Victoria | Australia |
| 243 | 05/13/08 | M | domestic | Queanbeyan | New South Wales | Australia |
| 244 | 03/28/08 | M | domestic | Auckland | North Island | NZ |
| 245 | 05/13/08 | M | domestic | Charnwood | ACT | Australia |
| 246 | 05/08/08 | F | domestic | Hastings | Victoria | Australia |
| 247 | 05/08/08 | M | domestic | Melbourne | Victoria | Australia |
| 249 | 05/08/08 | M | domestic | Melbourne | Victoria | Australia |
| 250 | 05/12/08 | M | domestic | Sydney | New South Wales | Australia |
| 252 | 04/21/08 | M | domestic | Middlemarch | South Island | NZ |
| 253 | 04/21/08 | M | domestic | Middlemarch | South Island | NZ |
| 254 | 04/21/08 | F | domestic | Middlemarch | South Island | NZ |
| 255 | 05/08/08 | F | domestic | Melbourne | Victoria | Australia |
| 256 | 05/12/08 | M | domestic | St. Mary's | New South Wales | Australia |
| 257 | 05/08/08 | F | domestic | Watsonia | Victoria | Australia |
| 259 | 05/08/08 | M | domestic | Bundoora | Victoria | Australia |
| 260 | 05/08/08 | F | domestic | Melbourne | Victoria | Australia |
| 261 | 05/09/08 | F | domestic | Auckland | North Island | NZ |
| 262 | 05/10/08 | M | domestic | Bombay | North Island | NZ |
| 263 | 05/12/08 | F | domestic | Sydney | New South Wales | Australia |
| 264 | 05/12/08 | F | domestic | Sydney | New South Wales | Australia |
| 265 | 05/12/08 | M | domestic | Padstow | New South Wales | Australia |
| 266 | 04/01/08 | F | domestic | Rangitaiki | North Island | NZ |
| 267 | 04/01/08 | M | domestic | Rangitaiki | North Island | NZ |
| 268 | 03/28/08 | M | domestic | Auckland | North Island | NZ |
| 269 | 03/28/08 | F | domestic | St. Stephens Village | North Island | NZ |
| 270 | 04/01/08 | M | domestic | Rangitaiki | North Island | NZ |
| 271 | 03/28/08 | F | domestic | Auckland | North Island | NZ |
| 272 | 03/28/08 | F | domestic | Cambridge | North Island | NZ |
| 273 | 03/28/08 | F | domestic | Cambridge | North Island | NZ |
| 274 | 05/12/08 | F | domestic | Kingswood | New South Wales | Australia |
| 275 | 05/08/08 | F | domestic | Hoppers Crossing | Victoria | Australia |
| 276 | 05/12/08 | M | domestic | Sydney | New South Wales | Australia |
| 277 | 05/12/08 | M | domestic | Sydney | New South Wales | Australia |
| 278 | 05/08/08 | F | domestic | Melbourne | Victoria | Australia |
| 279 | 05/08/08 | M | domestic | Melbourne | Victoria | Australia |
| 280 | 05/12/08 | M | domestic | South Penrith | New South Wales | Australia |
| 281 | 03/28/08 | M | domestic | Auckland | North Island | NZ |
| 282 | 05/12/08 | F | domestic | Sydney | New South Wales | Australia |
| 284 | 05/13/08 | M | domestic | Charnwood | ACT | Australia |
| 285 | 03/28/08 | M | domestic | Auckland | North Island | NZ |
| 286 | 05/08/08 | M | domestic | Hastings | Victoria | Australia |
| 287 | 05/13/08 | F | domestic | Dunlop | ACT | Australia |
| 288 | 05/13/08 | F | domestic | Charnwood | ACT | Australia |
| 289 | 05/19/08 | M | domestic | Greenwood | Western Australia | Australia |
| 292 | 05/13/08 | M | domestic | Mitchell | ACT | Australia |
| 293 | 05/13/08 | M | domestic | Kambah | ACT | Australia |
| 295 | 05/12/08 | F | domestic | Springwood | New South Wales | Australia |
| 296 | 05/13/08 | F | domestic | Kambah | ACT | Australia |
| 297 | 05/17/08 | M | domestic | Narangba | Queensland | Australia |
| 298 | 05/12/08 | F | domestic | Sydney | New South Wales | Australia |
| 300 | 05/13/08 | F | domestic | Mitchell | ACT | Australia |
| 303 | 05/13/08 | M | domestic | Torrens | ACT | Australia |
| 305 | 05/12/08 | M | domestic | Sydney | New South Wales | Australia |
| 306 | 05/13/08 | ? | domestic | South Brisbane | Queensland | Australia |
| 307 | 05/12/08 | M | domestic | Sydney | New South Wales | Australia |
| 308 | 05/01/08 | F | domestic | Brisbane | Queensland | Australia |
| 309 | 05/01/08 | F | domestic | Brisbane | Queensland | Australia |
| 310 | 05/12/08 | F | domestic | Sydney | New South Wales | Australia |
| 311 | 03/28/08 | F | domestic | Bombay | North Island | NZ |
| 312 | 05/13/08 | M | domestic | Ainslie | ACT | Australia |
| 313 | 05/19/08 | F | domestic | Greenwood | Western Australia | Australia |
| 314 | 05/13/08 | M | domestic | Fraser | ACT | Australia |
| 315 | 05/19/08 | F | domestic | Greenwood | Western Australia | Australia |
| 317 | 05/12/08 | M | domestic | Glenmore Park | New South Wales | Australia |
| 318 | 05/18/08 | M | domestic | Macleay Island | Queensland | Australia |
| 319 | 03/28/08 | M | domestic | Bombay | North Island | NZ |
| 320 | 05/12/08 | F | domestic | Springwood | New South Wales | Australia |
| 321 | 05/12/08 | F | domestic | South Penrith | New South Wales | Australia |
| 322 | 05/12/08 | F | domestic | South Penrith | New South Wales | Australia |
| 324 | 05/12/08 | M | domestic | South Penrith | New South Wales | Australia |
| 325 | 04/22/08 | F | domestic | Middlemarch | South Island | NZ |
| 326 | 04/21/08 | M | domestic | Middlemarch | South Island | NZ |
| 327 | 05/12/08 | M | domestic | Sydney | New South Wales | Australia |
| 328 | 05/12/08 | F | domestic | Glenmore Park | New South Wales | Australia |
| 329 | 05/13/08 | M | domestic | Charnwood | ACT | Australia |
| 330 | 05/08/08 | F | domestic | Hoppers Crossing | Victoria | Australia |
| 331 | 05/08/08 | F | domestic | Geelong | Victoria | Australia |
| 333 | 05/12/08 | M | domestic | Mulgoa | New South Wales | Australia |
| 335 | 05/08/08 | M | domestic | Melbourne | Victoria | Australia |
| 338 | 04/23/08 | M | domestic | Middlemarch | South Island | NZ |
| 339 | 05/07/08 | F | domestic | Risden Cove | Tasmania | Australia |
| 340 | 05/08/08 | F | domestic | Melbourne | Victoria | Australia |
| 341 | 05/08/08 | ? | domestic | Prahan | Victoria | Australia |
| 344 | 05/08/08 | F | domestic | Hastings | Victoria | Australia |
| 345 | 04/04/08 | M | domestic | Wanganui | North Island | NZ |
| 347 | 05/12/08 | M | domestic | St. Mary's | New South Wales | Australia |
| 348 | 05/08/08 | F | domestic | Watsonia | Victoria | Australia |
| 349 | 04/21/08 | F | domestic | Middlemarch | South Island | NZ |
| 352 | 05/08/08 | M | domestic | Hastings | Victoria | Australia |
| 354 | 05/13/08 | M | domestic | Charnwood | ACT | Australia |
| 355 | 05/07/08 | F | domestic | Risden Cove | Tasmania | Australia |
| 359 | 05/07/08 | F | domestic | Risden Cove | Tasmania | Australia |
| 362 | 05/08/08 | M | domestic | Melbourne | Victoria | Australia |
| 363 | 03/28/08 | M | domestic | Cambridge | North Island | NZ |
| 364 | 04/01/08 | M | domestic | Riparon | North Island | NZ |
| 367 | 04/01/08 | F | domestic | Riparon | North Island | NZ |
| 369 | 03/28/08 | F | domestic | Bombay | North Island | NZ |
| 371 | 04/01/08 | F | domestic | Rangitaiki | North Island | NZ |
| 373 | 04/01/08 | M | domestic | Riparon | North Island | NZ |
| 374 | 05/08/08 | M | domestic | Hoppers Crossing | Victoria | Australia |
| 380 | 05/08/08 | M | domestic | Melbourne | Victoria | Australia |
| 382 | 05/12/08 | F | domestic | Penrith | New South Wales | Australia |
| 385 | 05/08/08 | F | domestic | Melbourne | Victoria | Australia |
| 386 | 05/12/08 | F | domestic | Glenmore Park | New South Wales | Australia |
| 390 | 04/01/08 | F | domestic | Rangitaiki | North Island | NZ |
| 395 | 04/21/08 | F | domestic | Middlemarch | South Island | NZ |
| 396 | 04/21/08 | F | domestic | Middlemarch | South Island | NZ |
| 399 | 05/08/08 | M | domestic | Geelong | Victoria | Australia |
| 400 | 05/08/08 | F | domestic | Melbourne | Victoria | Australia |
| 401 | 05/08/08 | F | domestic | Melbourne | Victoria | Australia |
| 402 | 04/21/08 | M | domestic | Middlemarch | South Island | NZ |
| 403 | 05/12/08 | M | domestic | Werrington Downs | New South Wales | Australia |
| 404 | 04/21/08 | F | domestic | Middlemarch | South Island | NZ |
| 406 | 05/08/08 | F | domestic | Doveton | Victoria | Australia |
| 407 | 04/23/08 | M | domestic | Middlemarch | South Island | NZ |
| 410 | 05/19/08 | M | domestic | Greenwood | Western Australia | Australia |
| 412 | 05/08/08 | F | domestic | Melbourne | Victoria | Australia |
| 413 | 04/23/08 | M | domestic | Middlemarch | South Island | NZ |
| 416 | 05/08/08 | M | domestic | Briar Hill | Victoria | Australia |
| 420 | 05/08/08 | F | domestic | Doveton | Victoria | Australia |
| 421 | 05/12/08 | M | domestic | Sydney | New South Wales | Australia |
| 422 | 04/21/08 | M | domestic | Middlemarch | South Island | NZ |
| 423 | 05/08/08 | F | domestic | Melbourne | Victoria | Australia |
| 424 | 05/12/08 | M | domestic | St. Mary's | New South Wales | Australia |
| 425 | 04/21/08 | M | domestic | Middlemarch | South Island | NZ |
| 427 | 04/21/08 | M | domestic | Middlemarch | South Island | NZ |
| 428 | 05/08/08 | M | domestic | Hoppers Crossing | Victoria | Australia |
| 431 | 05/08/08 | ? | domestic | Prahan | Victoria | Australia |
| 432 | 04/23/08 | M | domestic | Middlemarch | South Island | NZ |
| 433 | 05/08/08 | F | domestic | Geelong | Victoria | Australia |
| 435 | 05/12/08 | F | domestic | Padstow | New South Wales | Australia |
| 440 | 05/13/08 | M | domestic | Fraser | ACT | Australia |
| 442 | 05/07/08 | M | domestic | Risden Cove | Tasmania | Australia |
| 443 | 05/08/08 | F | domestic | Hastings | Victoria | Australia |
| 446 | 05/07/08 | M | domestic | Upper Blessington | Tasmania | Australia |
| 447 | 05/08/08 | F | domestic | Melbourne | Victoria | Australia |
| 448 | 04/22/08 | M | domestic | Middlemarch | South Island | NZ |
| 449 | 05/08/08 | M | domestic | Doveton | Victoria | Australia |
| 450 | 04/21/08 | F | domestic | Middlemarch | South Island | NZ |
| 451 | 04/21/08 | M | domestic | Middlemarch | South Island | NZ |
| 453 | 05/07/08 | F | domestic | Risden Cove | Tasmania | Australia |
| 455 | 05/12/08 | M | domestic | South Penrith | New South Wales | Australia |
| 456 | 05/08/08 | F | domestic | Doveton | Victoria | Australia |
| 457 | 05/08/08 | F | domestic | Doveton | Victoria | Australia |
| 459 | 05/12/08 | M | domestic | Glenmore Park | New South Wales | Australia |
| 460 | 05/12/08 | M | domestic | Mulgoa | New South Wales | Australia |
| 461 | 05/08/08 | M | domestic | Melbourne | Victoria | Australia |
| 462 | 04/21/08 | M | domestic | Middlemarch | South Island | NZ |
| 464 | 05/08/08 | F | domestic | Melbourne | Victoria | Australia |
| 466 | 05/13/08 | M | domestic | Charnwood | ACT | Australia |
| 470 | 05/12/08 | M | domestic | Padstow | New South Wales | Australia |
| 477 | 05/08/08 | M | domestic | Rowville | Victoria | Australia |
| 480 | 05/13/08 | M | domestic | Queanbeyan | New South Wales | Australia |
| 482 | 05/08/08 | M | domestic | Mornington | Tasmania | Australia |
| 483 | 05/08/08 | F | domestic | Geelong | Victoria | Australia |
| 484 | 05/08/08 | F | domestic | Bayswater | Western Australia | Australia |
| 485 | 05/08/08 | M | domestic | Doveton | Victoria | Australia |
| 486 | 04/21/08 | F | domestic | Middlemarch | South Island | NZ |
| 488 | 05/07/08 | M | domestic | Risden Cove | Tasmania | Australia |
| 489 | 04/21/08 | F | domestic | Middlemarch | South Island | NZ |
| 490 | 05/08/08 | F | domestic | Geelong | Victoria | Australia |
| 491 | 05/08/08 | F | domestic | Doveton | Victoria | Australia |
| 494 | 04/04/08 | M | domestic | Wanganui | North Island | NZ |
| 495 | 05/12/08 | F | domestic | Penrith | New South Wales | Australia |
| 497 | 05/08/08 | F | domestic | Hastings | Victoria | Australia |
| 498 | 05/08/08 | F | domestic | Melbourne | Victoria | Australia |
| 499 | 04/21/08 | F | domestic | Middlemarch | South Island | NZ |
| 500 | 04/23/08 | M | domestic | Middlemarch | South Island | NZ |
| 501 | 06/15/08 | F | domestic | Bristol | South West England | England |
| 502 | 06/09/08 | M | domestic | Lenham | South East England | England |
| 503 | 06/10/08 | F | domestic | Lenham | South East England | England |
| 504 | 08/01/08 | M | domestic | Copenhagen | Hovedstaden | Denmark |
| 505 | 07/13/08 | M | domestic | Manchester | North West England | England |
| 506 | 08/01/08 | M | domestic | Copenhagen | Hovedstaden | Denmark |
| 508 | 06/11/08 | F | domestic | Wolverhampton | West midlands | England |
| 509 | 07/01/08 | F | domestic | Lelystad | Flavoland | Netherlands |
| 510 | 06/15/08 | M | domestic | Bristol | South West England | England |
| 511 | 06/10/08 | M | domestic | Lenham | South East England | England |
| 512 | 06/10/08 | M | domestic | Lenham | South East England | England |
| 513 | 07/13/08 | M | domestic | Manchester | North West England | England |
| 514 | 06/13/08 | F | domestic | Edinburgh |  | Scotland |
| 515 | 06/15/08 | M | domestic | Gloucester | South West England | England |
| 516 | 06/16/08 | F | domestic | Gravesend | South East England | ENGsp |
| 517 | 06/16/08 | F | domestic | Gravesend | South East England | ENGsp |
| 518 | 06/13/08 | F | domestic | Edinburgh |  | Scotland |
| 519 | 07/13/08 | F | domestic | Manchester | North West England | England |
| 520 | 07/13/08 | M | polecat | Manchester | North West England | England |
| 521 | 08/01/08 | F | domestic | Fredricksberg | Hovedstaden | Denmark |
| 522 | 08/01/08 | M | domestic | Bronshoj | Hovedstaden | Denmark |
| 523 | 06/15/08 | M | domestic | Hengrove/Bristol | South West England | England |
| 524 | 06/11/08 | M | domestic | Wolverhampton | West midlands | England |
| 525 | 08/01/08 | M | domestic | Visby | Gotlands Lan | Sweden |
| 526 | 07/13/08 | F | domestic | Manchester | North West England | England |
| 527 | 06/11/08 | M | domestic | Wolverhampton | West midlands | England |
| 528 | 06/11/08 | F | domestic | Wolverhampton | West midlands | England |
| 529 | 06/11/08 | M | domestic | Wolverhampton | West midlands | England |
| 530 | 06/14/08 | M | domestic | Bestwood Park | East midlands | England |
| 531 | 07/13/08 | F | domestic | Manchester | North West England | England |
| 533 | 08/01/08 | M | domestic | Hasselby | Stockholms Lan | Sweden |
| 534 | 06/10/08 | M | domestic | Lenham | South East England | England |
| 535 | 06/11/08 | M | domestic | Telford | West midlands | England |
| 536 | 06/11/08 | M | domestic | Wolverhampton | West midlands | England |
| 537 | 06/12/08 | M | domestic | Bathgate | Scotland | Scotland |
| 538 | 07/13/08 | F | domestic | Manchester | North West England | England |
| 539 | 06/10/08 | M | domestic | Lenham | South East England | England |
| 540 | 08/01/08 | M | domestic | Copenhagen | Hovedstaden | Denmark |
| 541 | 06/15/08 | M | domestic | Marshfield | South West England | England |
| 542 | 06/14/08 | M | domestic | Bestwood Park | East midlands | England |
| 543 | 08/01/08 | F | domestic | Budapest | Budapest | Hungary |
| 544 | 08/01/08 | M | domestic | Bronshoj | Hovedstaden | Denmark |
| 545 | 06/10/08 | M | domestic | Lenham | South East England | England |
| 547 | 06/08/08 | F | domestic | Lenham | South East England | England |
| 548 | 06/06/08 | F | domestic | Lenham | South East England | England |
| 550 | 08/01/08 | F | domestic | Valby | Hovedstaden | Denmark |
| 551 | 06/13/08 | M | domestic | Edinburgh |  | Scotland |
| 552 | 06/10/08 | F | domestic | Sittingbourne | South East England | England |
| 553 | 06/14/08 | M | domestic | Carlton | East midlands | England |
| 555 | 08/01/08 | M | domestic | Ranas | Stockholms Lan | Sweden |
| 556 | 06/10/08 | F | domestic | Lenham | South East England | England |
| 557 | 06/10/08 | M | domestic | Lenham | South East England | England |
| 559 | 08/01/08 | M | domestic | Fredricksberg | Hovedstaden | Denmark |
| 560 | 06/16/08 | M | domestic | Gravesend | South East England | ENGsp |
| 561 | 06/11/08 | F | domestic | Wolverhampton | West midlands | England |
| 562 | 06/11/08 | M | domestic | Telford | West midlands | England |
| 563 | 06/11/08 | M | domestic | Wolverhampton | West midlands | England |
| 564 | 07/13/08 | M | domestic | Denton | North West England | England |
| 565 | 07/13/08 | M | domestic | Denton | North West England | England |
| 567 | 06/13/08 | F | domestic | Edinburgh |  | Scotland |
| 568 | 07/13/08 | F | domestic | Denton | North West England | England |
| 569 | 06/14/08 | F | domestic | Carlton | East midlands | England |
| 571 | 06/15/08 | F | domestic | Bristol | South West England | England |
| 572 | 07/13/08 | F | domestic | Denton | North West England | England |
| 573 | 08/01/08 | F | domestic | Fredricksberg | Hovedstaden | Denmark |
| 574 | 06/15/08 | F | domestic | Gloucester | South West England | England |
| 575 | 07/13/08 | M | domestic | Denton | North West England | England |
| 580 | 06/10/08 | F | domestic | Sittingbourne | South East England | England |
| 581 | 06/09/08 | F | domestic | Lenham | South East England | England |
| 582 | 08/01/08 | F | domestic | Katrineholm | Sodermanlands Lan | Sweden |
| 583 | 07/13/08 | M | domestic | Denton | North West England | England |
| 584 | 07/13/08 | M | domestic | Manchester | North West England | England |
| 585 | 08/01/08 | M | domestic | Fredricksberg | Hovedstaden | Denmark |
| 588 | 06/10/08 | F | domestic | Lenham | South East England | England |
| 589 | 08/01/08 | F | domestic | Fredricksberg | Hovedstaden | Denmark |
| 590 | 08/01/08 | M | domestic | Ljugarn | Gotlands Lan | Sweden |
| 591 | 07/13/08 | F | domestic | Manchester | North West England | England |
| 592 | 08/01/08 | M | domestic | Ljugarn | Gotlands Lan | Sweden |
| 593 | 06/10/08 | M | domestic | Lenham | England | England |
| 596 | 07/13/08 | M | domestic | Manchester | North West England | England |
| 597 | 06/12/08 | F | domestic | Bathgate | Scotland | Scotland |
| 605 | 06/16/08 | F | domestic | Gravesend | South East England | ENGsp |
| 610 | 08/01/08 | M | domestic | Orebro | Orebro Lan | Sweden |
| 612 | 07/01/08 | F | domestic | Lelystad | Flavoland | Netherlands |
| 626 | 08/01/08 | F | domestic | Ljugarn | Gotlands Lan | Sweden |
| 632 | 08/01/08 | F | domestic | Hasselby | Stockholms Lan | Sweden |
| 640 | 08/01/08 | M | domestic | Hasselby | Stockholms Lan | Sweden |
| 641 | 06/16/08 | M | domestic | Gravesend | South East England | ENGsp |
| 642 | 08/01/08 | M | domestic | Copenhagen | Hovedstaden | Denmark |
| 645 | 06/13/08 | M | domestic | Edinburgh |  | Scotland |
| 646 | 06/12/08 | F | domestic | Bathgate | Scotland | Scotland |
| 647 | 08/01/08 | F | domestic | Hasselby | Stockholms Lan | Sweden |
| 648 | 06/16/08 | F | domestic | Gravesend | South East England | ENGsp |
| 651 | 08/01/08 | F | domestic | Tastrup | Hovedstaden | Denmark |
| 654 | 08/01/08 | F | domestic | Ljugarn | Gotlands Lan | Sweden |
| 659 | 08/01/08 | M | domestic | Hasselby | Stockholms Lan | Sweden |
| 662 | 06/16/08 | M | domestic | Gravesend | South East England | ENGsp |
| 663 | 08/01/08 | F | domestic | Copenhagen | Hovedstaden | Denmark |
| 672 | 08/01/08 | M | domestic | Fredricksberg | Hovedstaden | Denmark |
| 675 | 06/13/08 | M | domestic | Edinburgh |  | Scotland |
| 677 | 08/01/08 | F | domestic | Ranas | Stockholms Lan | Sweden |
| 678 | 06/16/08 | F | domestic | Gravesend | South East England | ENGsp |
| 680 | 06/16/08 | F | domestic | Gravesend | South East England | ENGsp |
| 681 | 06/16/08 | F | domestic | Gravesend | South East England | ENGsp |
| 682 | 08/01/08 | M | domestic | Bronshoj | Hovedstaden | Denmark |
| 683 | 08/01/08 | M | domestic | Visby | Gotlands Lan | Sweden |
| 684 | 08/01/08 | F | domestic | Visby | Gotlands Lan | Sweden |
| 690 | 08/01/08 | F | domestic | Holte | Hovedstaden | Denmark |
| 691 | 06/12/08 | F | domestic | Bathgate | Scotland | Scotland |
| 695 | 08/01/08 | M | domestic | Katrineholm | Sodermanlands Lan | Sweden |
| 697 | 08/01/08 | F | domestic | Ljugarn | Gotlands Lan | Sweden |
| 703 | 08/01/08 | F | domestic | Orebro | Orebro Lan | Sweden |
| 704 | 08/01/08 | F | domestic | Ljugarn | Gotlands Lan | Sweden |
| 706 | 08/01/08 | F | domestic | Orebro | Orebro Lan | Sweden |
| 710 | 07/01/08 | M | domestic | Lelystad | Flavoland | Netherlands |
| 711 | 06/12/08 | F | domestic | Bathgate | Scotland | Scotland |
| 713 | 08/01/08 | M | domestic | Ljugarn | Gotlands Lan | Sweden |
| 715 | 08/01/08 | M | domestic | Tingstade | Gotlands Lan | Sweden |
| 719 | 08/01/08 | F | domestic | Fredricksberg | Hovedstaden | Denmark |
| 720 | 06/13/08 | M | domestic | Edinburgh |  | Scotland |
| 722 | 08/01/08 | M | domestic | Holte | Hovedstaden | Denmark |
| 723 | 06/13/08 | M | domestic | Edinburgh |  | Scotland |
| 726 | 08/01/08 | M | domestic | Budapest | Budapest | Hungary |
| 731 | 08/01/08 | F | domestic | Ljugarn | Gotlands Lan | Sweden |
| 733 | 06/12/08 | M | domestic | Bathgate | Scotland | Scotland |
| 734 | 06/12/08 | M | domestic | Bathgate | Scotland | Scotland |
| 735 | 06/10/08 | M | polecat |  |  | Wales |
| 737 | 08/01/08 | F | domestic | Copenhagen | Hovedstaden | Denmark |
| 738 | 06/12/08 | F | domestic | Bathgate | Scotland | Scotland |
| 739 | 08/01/08 | F | domestic | Copenhagen | Hovedstaden | Denmark |
| 741 | 08/01/08 | F | domestic | Tingstade | Gotlands Lan | Sweden |
| 742 | 08/01/08 | M | domestic | Valby | Hovedstaden | Denmark |
| 743 | 08/01/08 | M | domestic | Fredricksberg | Hovedstaden | Denmark |
| 746 | 08/01/08 | M | domestic | Bronshoj | Hovedstaden | Denmark |
| 747 | 08/01/08 | M | domestic | Holte | Hovedstaden | Denmark |
| 749 | 08/01/08 | F | domestic | Fredricksberg | Hovedstaden | Denmark |
| 758 | 08/01/08 | M | domestic | Bergen | Hordaland | Norway |
| 759 | 08/01/08 | F | domestic | Florvag | Hordaland | Norway |
| 760 | 08/01/08 | F | domestic | Bergen | Hordaland | Norway |
| 761 | 08/01/08 | F | domestic | Florvag | Hordaland | Norway |
| 762 | 08/01/08 | F | domestic | Bergen | Hordaland | Norway |
| 763 | 08/01/08 | M | domestic | Trondheim | Sor-Trondelag | Norway |
| 764 | 08/01/08 | F | domestic | Trondheim | Sor-Trondelag | Norway |
| 765 | 08/01/08 | M | domestic | Saupstad | Sor-Trondelag | Norway |
| 766 | 08/01/08 | M | domestic |  | Sor-Trondelag | Norway |
| 771 | 08/01/08 | M | domestic | Saupstad | Sor-Trondelag | Norway |
| 773 | 08/01/08 | F | domestic | Trondheim | Sor-Trondelag | Norway |
| 774 | 08/01/08 | M | domestic | Trondheim | Sor-Trondelag | Norway |
| 778 | 09/01/08 | M | domestic | Columbia | MO | USA |
| 779 | 10/01/08 | F | domestic | Columbia | MO | USA |
| 780 | 08/01/08 | F | domestic | Trondheim | Sor-Trondelag | Norway |
| 781 | 08/01/08 | M | domestic | Saupstad | Sor-Trondelag | Norway |
| 782 | 08/17/08 | F | domestic | Almere-Buiten | Flavoland | Netherlands |
| 783 | 08/17/08 | M | domestic | Bilthoven | Utrecht | Netherlands |
| 785 | 09/01/08 | M | domestic | Marengo | OH | USA |
| 786 | 09/01/08 | F | domestic | Columbia | MO | USA |
| 787 | 10/01/08 | M | domestic | Columbia | MO | USA |
| 791 | 08/01/08 | M | domestic | Odense | Syddanmark | Denmark |
| 792 | 08/01/08 | M | domestic | Ljugarn | Gotland | Sweden |
| 793 | 08/01/08 | F | domestic | Odense | Syddanmark | Denmark |
| 794 | 08/01/08 | M | domestic | Saupstad | Sor-Trondelag | Norway |
| 795 | 08/01/08 | F | domestic |  | Sor-Trondelag | Norway |
| 796 | 08/01/08 | F | domestic | Trondheim | Sor-Trondelag | Norway |
| 797 | 08/01/08 | M | domestic | Trondheim | Sor-Trondelag | Norway |
| 798 | 08/01/08 | M | domestic |  | Sor-Trondelag | Norway |
| 799 | 08/01/08 | F | domestic | Trondheim | Sor-Trondelag | Norway |
| 800 | 09/01/08 | F | domestic | Columbia | MO | USA |
| 803 | 10/01/08 | M | domestic | St. Louis | MO | USA |
| 804 | 10/01/08 | M | domestic | St. Louis | MO | USA |
| 809 | 08/01/08 | F | domestic | Florvag | Hordaland | Norway |
| 810 | 08/01/08 | F | domestic | Assens | Nordjylland | Denmark |
| 811 | 08/01/08 | F | domestic | Trondheim | Sor-Trondelag | Norway |
| 812 | 08/01/08 | F | domestic | Assens | Nordjylland | Denmark |
| 813 | 08/01/08 | M | domestic | Saupstad | Sor-Trondelag | Norway |
| 814 | 08/01/08 | F | domestic | Trondheim | Sor-Trondelag | Norway |
| 815 | 08/01/08 | F | domestic | Trondheim | Sor-Trondelag | Norway |
| 817 | 07/01/08 | F | domestic | Almere-Buiten | Flavoland | Netherlands |
| 819 | 10/01/08 | F | domestic | Columbia | MO | USA |
| 820 | 08/01/08 | M | domestic | Budapest | Budapest | Hungary |
| 823 | 10/01/08 | M | domestic | Columbia | MO | USA |
| 828 | 08/01/08 | F | domestic | Budapest | Budapest | Hungary |
| 830 | 10/01/08 | M | domestic | Columbia | MO | USA |
| 831 | 08/01/08 | F | domestic | Lelystad | Flavoland | Netherlands |
| 832 | 08/01/08 | M | domestic | Lelystad | Flavoland | Netherlands |
| 833 | 08/01/08 | M | domestic | Lelystad | Flavoland | Netherlands |
| 834 | 08/01/08 | F | domestic | Lelystad | Flavoland | Netherlands |
| 843 | 10/01/08 | F | domestic | St. Louis | MO | USA |
| 844 | 09/01/08 | F | domestic | Columbia | MO | USA |
| 845 | 08/01/08 | M | domestic | Lelystad | Flavoland | Netherlands |
| 847 | 08/01/08 | F | domestic | Budapest | Budapest | Hungary |
| 849 | 08/01/08 | F | domestic | Budapest | Budapest | Hungary |
| 850 | 08/01/08 | F | domestic |  | Sor-Trondelag | Norway |
| 851 | 08/01/08 | F | domestic |  | Sor-Trondelag | Norway |
| 852 | 08/01/08 | M | domestic | Odense | Syddanmark | Denmark |
| 853 | 08/01/08 | F | domestic | Vodskov | Nordjylland | Denmark |
| 856 | 08/01/08 | F | domestic | Budapest | Budapest | Hungary |
| 857 | 10/01/08 | F | domestic | Columbia | MO | USA |
| 862 | 08/01/08 | F | domestic | Florvag | Hordaland | Norway |
| 863 | 08/01/08 | F | domestic | Assens | Nordjylland | Denmark |
| 864 | 08/01/08 | F | domestic | Bergen | Hordaland | Norway |
| 865 | 08/01/08 | F | domestic | Florvag | Hordaland | Norway |
| 866 | 08/01/08 | F | domestic | Assens | Nordjylland | Denmark |
| 867 | 08/01/08 | F | domestic |  | Sor-Trondelag | Norway |
| 868 | 08/01/08 | M | domestic | Florvag | Hordaland | Norway |
| 869 | 08/01/08 | M | domestic | Assens | Nordjylland | Denmark |
| 870 | 10/01/08 | M | domestic | St. Louis | MO | USA |
| 871 | 10/01/08 | M | domestic | St. Louis | MO | USA |
| 872 | 09/01/08 | F | domestic | Columbia | MO | USA |
| 873 | 09/01/08 | M | domestic | Columbia | MO | USA |
| 874 | 10/01/08 | F | domestic | Columbia | MO | USA |
| 875 | 09/01/08 | M | domestic | Columbia | MO | USA |
| 876 | 09/01/08 | F | domestic | Columbia | MO | USA |
| 877 | 10/01/08 | M | domestic | Columbia | MO | USA |
| 878 | 08/01/08 | M | domestic | Vodskov | Nordjylland | Denmark |
| 879 | 08/01/08 | M | domestic | Budapest | Budapest | Hungary |
| 880 | 08/01/08 | F | domestic | Vodskov | Nordjylland | Denmark |
| 883 | 08/01/08 | M | domestic | Budapest | Budapest | Hungary |
| 884 | 08/01/08 | M | domestic | Budapest | Budapest | Hungary |
| 885 | 08/01/08 | F | domestic | Budapest | Budapest | Hungary |
| 886 | 08/01/08 | F | domestic | Budapest | Budapest | Hungary |
| 887 | 08/01/08 | M | domestic | Lelystad | Flavoland | Netherlands |
| 888 | 08/01/08 | M | domestic | Lelystad | Flavoland | Netherlands |
| 889 | 08/01/08 | F | domestic | Lelystad | Flavoland | Netherlands |
| 892 | 10/01/08 | F | domestic | Columbia | MO | USA |
| 893 | 10/01/08 | F | domestic | Columbia | MO | USA |
| 894 | 10/01/08 | F | domestic | Columbia | MO | USA |
| 896 | 08/01/08 | M | domestic | Vodskov | Nordjylland | Denmark |
| 897 | 07/01/08 | F | domestic | Almere-Buiten | Flavoland | Netherlands |
| 898 | 08/01/08 | M | domestic | Budapest | Budapest | Hungary |
| 899 | 08/01/08 | F | domestic | Budapest | Budapest | Hungary |
| 900 | 08/01/08 | F | domestic | Odense | Syddanmark | Denmark |
| 901 | 08/01/08 | F | domestic | Frederikshavn | Nordjylland | Denmark |
| 902 | 08/01/08 | M | domestic |  | Sor-Trondelag | Norway |
| 903 | 08/01/08 | M | domestic |  | Sor-Trondelag | Norway |
| 905 | 08/01/08 | M | domestic | Budapest | Budapest | Hungary |
| 906 | 08/01/08 | M | domestic | Silkeborg | Midtjylland | Denmark |
| 907 | 10/01/08 | F | domestic | Columbia | MO | USA |
| 908 | 08/17/08 | F | domestic | Bilthoven | Utrecht | Netherlands |
| 909 | 08/17/08 | M | domestic | Bilthoven | Utrecht | Netherlands |
| 910 | 08/17/08 | M | domestic | Bilthoven | Utrecht | Netherlands |
| 913 | 08/01/08 | M | domestic | Lelystad | Flavoland | Netherlands |
| 914 | 08/01/08 | M | domestic | Lelystad | Flavoland | Netherlands |
| 915 | 08/01/08 | F | domestic | Lelystad | Flavoland | Netherlands |
| 917 | 08/17/08 | F | domestic | Bilthoven | Utrecht | Netherlands |
| 918 | 08/01/08 | F | domestic | Budapest | Budapest | Hungary |
| 919 | 07/01/08 | M | domestic | Almere-Buiten | Flavoland | Netherlands |
| 924 | 07/07/08 | F | domestic | Almere-Buiten | Flavoland | Netherlands |
| 925 | 07/01/08 | M | domestic | Almere-Buiten | Flavoland | Netherlands |
| 933 | 08/01/08 | F | domestic | Lelystad | Flavoland | Netherlands |
| 934 | 08/01/08 | F | domestic | Lelystad | Flavoland | Netherlands |
| 935 | 08/01/08 | F | domestic | Lelystad | Flavoland | Netherlands |
| 936 | 08/01/08 | M | domestic | Lelystad | Flavoland | Netherlands |
| 937 | 08/01/08 | F | domestic | Lelystad | Flavoland | Netherlands |
| 938 | 08/17/08 | F | domestic | Bilthoven | Utrecht | Netherlands |
| 939 | 08/17/08 | F | domestic | Bilthoven | Utrecht | Netherlands |
| 940 | 08/01/08 | F | domestic | Lelystad | Flavoland | Netherlands |
| 941 | 08/01/08 | F | domestic | Lelystad | Flavoland | Netherlands |
| 942 | 08/01/08 | F | domestic | Lelystad | Flavoland | Netherlands |
| 943 | 08/01/08 | F | domestic | Lelystad | Flavoland | Netherlands |
| 945 | 08/01/08 | M | domestic | Assens | Nordjylland | Denmark |
| 946 | 08/01/08 | M | domestic | Bergen | Hordaland | Norway |
| 947 | 08/01/08 | M | domestic | Bergen | Hordaland | Norway |
| 948 | 08/01/08 | F | domestic | Frederikshavn | Nordjylland | Denmark |
| 949 | 08/01/08 | F | domestic |  | Sor-Trondelag | Norway |
| 950 | 08/01/08 | F | domestic |  | Sor-Trondelag | Norway |
| 951 | 08/01/08 | F | domestic | Trondheim | Sor-Trondelag | Norway |
| 952 | 08/01/08 | F | domestic | Trondheim | Sor-Trondelag | Norway |
| 953 | 08/01/08 | F | domestic | Budapest | Budapest | Hungary |
| 954 | 08/17/08 | F | domestic | Bilthoven | Utrecht | Netherlands |
| 955 | 08/17/08 | M | domestic | Budapest | Budapest | Hungary |
| 956 | 08/01/08 | F | domestic | Assens | Nordjylland | Denmark |
| 957 | 08/01/08 | F | domestic | Assens | Nordjylland | Denmark |
| 958 | 08/01/08 | F | domestic | Assens | Nordjylland | Denmark |
| 959 | 08/01/08 | M | domestic | Lelystad | Flavoland | Netherlands |
| 960 | 08/01/08 | M | domestic | Lelystad | Flavoland | Netherlands |
| 961 | 10/01/08 | M | domestic | St. Louis | MO | USA |
| 963 | 08/01/08 | F | domestic | Lelystad | Flavoland | Netherlands |
| 965 | 08/01/08 | M | domestic | Assens | Nordjylland | Denmark |
| 966 | 08/01/08 | M | domestic | Assens | Nordjylland | Denmark |
| 967 | 08/01/08 | M | domestic | Assens | Nordjylland | Denmark |
| 968 | 08/01/08 | M | domestic | Assens | Nordjylland | Denmark |
| 969 | 08/01/08 | M | domestic | Lelystad | Flavoland | Netherlands |
| 970 | 08/01/08 | F | domestic | Frederikshavn | Nordjylland | Denmark |
| 971 | 08/01/08 | M | domestic | Frederikshavn | Nordjylland | Denmark |
| 972 | 08/01/08 | F | domestic | Odense | Syddanmark | Denmark |
| 973 | 08/01/08 | M | domestic | Vodskov | Nordjylland | Denmark |
| 974 | 08/17/08 | M | domestic | Bilthoven | Utrecht | Netherlands |
| 975 | 07/01/08 | M | domestic | Almere-Buiten | Flavoland | Netherlands |
| 976 | 10/01/08 | M | domestic | St. Louis | MO | USA |
| 979 | 07/01/08 | M | domestic | Almere-Buiten | Flavoland | Netherlands |
| 981 | 08/17/08 | M | domestic | Almere-Buiten | Flavoland | Netherlands |
| 982 | 08/01/08 | F | domestic | Vodskov | Nordjylland | Denmark |
| 991 | 07/01/08 | F | domestic | Almere-Buiten | Flavoland | Netherlands |
| 992 | 10/01/08 | F | domestic | Columbia | MO | USA |
| 993 | 08/17/08 | M | domestic | Almere-Buiten | Flavoland | Netherlands |
| 994 | 08/01/08 | M | domestic | Frederikshavn | Nordjylland | Denmark |
| 996 | 08/17/08 | M | domestic | Bilthoven | Utrecht | Netherlands |
| 997 | 08/01/08 | M | domestic |  | Sor-Trondelag | Norway |
| 999 | 08/01/08 | M | domestic | Budapest | Budapest | Hungary |
| 1001 | 12/09/08 | F | domestic | El Cerrito | CA | USA |
| 1006 | 09/23/10 | M | polecat | Bristol | South West England | England |
| 1011 | 11/13/10 | M | polecat | Macclesfield | North West England | England |
| 1030 | 11/13/10 | M | polecat | Macclesfield | North West England | England |
| 1031 | 09/25/10 | M | polecat | Bristol | South West England | England |
| 1085 | 04/30/10 | F | domestic | Lynwood | WA | USA |
| 1086 | 04/30/10 | F | domestic | Kirkland | WA | USA |
| 1088 | 04/30/10 | M | domestic | Kirkland | WA | USA |
| 1089 | 04/30/10 | F | domestic | Bellevue | WA | USA |
| 1090 | 03/27/10 | F | domestic |  | NJ | USA |
| 1091 | 04/13/10 | M | domestic | Roseisle | Manitoba | Canada |
| 1093 | 04/30/10 | M | domestic | Seattle | WA | USAsp |
| 1094 | 04/30/10 | M | domestic | Seattle | WA | USAsp |
| 1095 | 04/30/10 | M | domestic | Seattle | WA | USAsp |
| 1096 | 04/30/10 | M | domestic | Kirkland | WA | USA |
| 1097 | 08/31/10 | M | domestic | West Kirkland | WA | USA |
| 1098 | 04/17/10 | F | domestic | Roseisle | Manitoba | Canada |
| 1099 | 04/03/10 | M | domestic | Kirkland | WA | USA |
| 1100 | 04/13/10 | M | domestic | Roseisle | Manitoba | Canada |
| 1101 | 04/03/10 | M | domestic | Roseisle | Manitoba | Canada |
| 1102 | 04/13/10 | F | domestic | Roseisle | Manitoba | Canada |
| 1103 | 04/13/10 | M | domestic | Roseisle | Manitoba | Canada |
| 1104 | 04/13/10 | M | domestic | Roseisle | Manitoba | Canada |
| 1105 | 04/08/10 | F | domestic | Kirkland | WA | USA |
| 1106 | 04/01/10 | M | domestic | Seattle | WA | USAsp |
| 1107 | 04/13/10 | M | domestic | Roseisle | Manitoba | Canada |
| 1108 | 04/13/10 | M | domestic | Roseisle | Manitoba | Canada |
| 1110 | 04/30/10 | F | domestic | Kirkland | WA | USA |
| 1111 | 04/03/10 | M | domestic | Roseisle | Manitoba | Canada |
| 1112 | 04/30/10 | M | domestic | Roseisle | Manitoba | Canada |
| 1113 | 04/06/10 | F | domestic | Roseisle | Manitoba | Canada |
| 1114 | 04/06/10 | M | domestic | Roseisle | Manitoba | Canada |
| 1115 | 04/06/10 | M | domestic | Roseisle | Manitoba | Canada |
| 1116 | 04/13/10 | M | domestic | Roseisle | Manitoba | Canada |
| 1117 | 04/13/10 | F | domestic | Roseisle | Manitoba | Canada |
| 1118 | 04/13/10 | M | domestic | Roseisle | Manitoba | Canada |
| 1119 | 04/13/10 | F | domestic | Roseisle | Manitoba | Canada |
| 1120 | 04/06/10 | F | domestic | Edmonds | WA | USA |
| 1121 | 04/03/10 | M | domestic | Roseisle | Manitoba | Canada |
| 1122 | 04/13/10 | M | domestic | Roseisle | Manitoba | Canada |
| 1123 | 04/13/10 | M | domestic | Roseisle | Manitoba | Canada |
| 1124 | 04/13/10 | M | domestic | Roseisle | Manitoba | Canada |
| 1125 | 04/13/10 | M | domestic | Roseisle | Manitoba | Canada |
| 1126 | 04/13/10 | F | domestic | Roseisle | Manitoba | Canada |
| 1127 | 04/13/10 | F | domestic | Roseisle | Manitoba | Canada |
| 1128 | 04/13/10 | F | domestic | Roseisle | Manitoba | Canada |
| 1129 | 04/26/10 | F | domestic | Maple Valley | WA | USA |
| 1130 | 04/13/10 | F | domestic | Roseisle | Manitoba | Canada |
| 1131 | 04/13/10 | M | domestic | Roseisle | Manitoba | Canada |
| 1132 | 04/13/10 | M | domestic | Roseisle | Manitoba | Canada |
| 1133 | 04/13/10 | F | domestic | Roseisle | Manitoba | Canada |
| 1134 | 04/13/10 | M | domestic | Roseisle | Manitoba | Canada |
| 1135 | 04/13/10 | F | domestic | Roseisle | Manitoba | Canada |
| 1136 | 04/13/10 | M | domestic | Roseisle | Manitoba | Canada |
| 1137 | 04/13/10 | M | domestic | Roseisle | Manitoba | Canada |
| 1138 | 04/13/10 | M | domestic | Roseisle | Manitoba | Canada |
| 1139 | 04/13/10 | F | domestic | Roseisle | Manitoba | Canada |
| 1140 | 04/13/10 | M | domestic | Roseisle | Manitoba | Canada |
| 1141 | 04/13/10 | M | domestic | Roseisle | Manitoba | Canada |
| 1142 | 01/25/11 | M | domestic | Edmonds | WA | USA |
| 1143 | 01/25/11 | F | domestic | Edmonds | WA | USA |
| 1144 | 04/30/10 | M | domestic | Bellevue | WA | USA |
| 1145 | 08/31/10 | F | domestic | West Kirkland | WA | USA |
| 1146 | 04/30/10 | M | domestic | Kirkland | WA | USA |
| 1147 | 04/13/10 | M | domestic | Roseisle | Manitoba | Canada |
| 1148 | 04/13/10 | F | domestic | Roseisle | Manitoba | Canada |
| 1149 | 04/13/10 | F | domestic | Roseisle | Manitoba | Canada |
| 1150 | 04/13/10 | F | domestic | Roseisle | Manitoba | Canada |
| 1151 | 04/13/10 | F | domestic | Roseisle | Manitoba | Canada |
| 1152 | 04/13/10 | F | domestic | Roseisle | Manitoba | Canada |
| 1153 | 01/25/11 | F | domestic | Edmonds | WA | USA |
| 1156 | 07/22/10 | F | domestic | Bothell | WA | USA |
| 1157 | 04/16/10 | M | domestic | Seattle | WA | USA |
| 1159 | 04/30/10 | F | domestic | Seattle | WA | USAsp |
| 1160 | 05/22/10 | F | domestic | Ashford | WA | USA |
| 1161 | 04/13/10 | M | domestic | Roseisle | Manitoba | Canada |
| 1162 | 04/13/10 | M | domestic | Roseisle | Manitoba | Canada |
| 1164 | 04/13/10 | M | domestic | Roseisle | Manitoba | Canada |
| 1165 | 04/13/10 | F | domestic | Roseisle | Manitoba | Canada |
| 1166 | 04/13/10 | M | domestic | Roseisle | Manitoba | Canada |
| 1167 | 04/13/10 | M | domestic | Roseisle | Manitoba | Canada |
| 1168 | 04/13/10 | F | domestic | Roseisle | Manitoba | Canada |
| 1169 | 04/13/10 | M | domestic | Roseisle | Manitoba | Canada |
| 1170 | 04/13/10 | F | domestic | Roseisle | Manitoba | Canada |
| 1171 | 04/13/10 | F | domestic | Roseisle | Manitoba | Canada |
| 1179 | 04/13/10 | M | domestic | Roseisle | Manitoba | Canada |
| 1180 | 06/12/10 | F | domestic | Bellevue | WA | USA |
| 1181 | 04/13/10 | M | domestic | Roseisle | Manitoba | Canada |
| 1183 | 08/31/10 | M | domestic | West Kirkland | WA | USA |
| 1194 | 08/31/10 | F | domestic |  | MD | USA |
| 1200 | 06/17/10 | F | domestic | Redmond | WA | USA |
| 1202 | 01/01/11 | M | polecat | Netheravon | South West England | England |
| 1203 | 01/01/11 | F | polecat | Netheravon | South West England | England |
| 1204 | 06/06/11 | F | Polecat | Newbury Berkshire | South East England | England |
| 1205 | 01/01/11 | F | polecat | Netheravon | South West England | England |
| 1212 | 01/01/11 | M | polecat | Netheravon | South West England | England |
| 1220 | 01/01/11 | M | polecat | Netheravon | South West England | England |
| 1225 | 06/12/11 | M | polecat | Newbury Berkshire | South East England | England |
| 1234 | 06/06/11 | F | polecat | Newbury Berkshire | South East England | England |
| 1248 | 06/06/11 | M | polecat | Newbury Berkshire | South East England | England |
| 1252 | 03/11/11 | F | domestic | Martinez | CA | USA |
| 1260 | 03/23/11 | M | domestic | Union City | CA | USA |
| 1265 | 03/07/11 | F | domestic | San Jose | CA | USA |
| 1268 | 03/11/11 | F | domestic | Martinez | CA | USA |
| 1271 | 03/23/11 | M | domestic | Union City | CA | USA |
| 1273 | 03/11/11 | M | domestic | Martinez | CA | USA |
| 1275 | 12/01/10 | M | domestic | Castro Valley | CA | USA |
| 1283 | 01/27/11 | F | domestic | Indianapolis | IN | USA |
| 1284 | 12/10/10 | F | domestic | Kokomo | IN | USA |
| 1285 | 12/13/10 | M | domestic | Zionsville | IN | USA |
| 1286 | 12/31/10 | M | domestic | Indianapolis | IN | USA |
| 1287 | 12/31/10 | M | domestic | Indianapolis | IN | USA |
| 1288 | 03/11/11 | M | domestic | Oregon City | OR | USA |
| 1289 | 03/05/11 | F | domestic | Portland | OR | USA |
| 1290 | 01/27/11 | F | domestic | Indianapolis | IN | USA |
| 1291 | 03/06/11 | F | domestic | Portland | OR | USA |
| 1292 | 12/31/10 | F | domestic | Indianapolis | IN | USA |
| 1293 | 12/31/10 | F | domestic | Indianapolis | IN | USA |
| 1294 | 12/31/10 | F | domestic | Indianapolis | IN | USA |
| 1296 | 01/27/11 | F | domestic | Indianapolis | IN | USA |
| 1297 | 01/27/11 | M | domestic | Indianapolis | IN | USA |
| 1301 | 12/31/10 | M | domestic | Indianapolis | IN | USA |
| 1302 | 12/31/10 | F | domestic | Indianapolis | IN | USA |
| 1303 | 12/31/10 | M | domestic | Indianapolis | IN | USA |
| 1304 | 12/31/10 | M | domestic | Indianapolis | IN | USA |
| 1308 | 12/31/10 | F | domestic | Indianapolis | IN | USA |
| 1311 | 12/31/10 | M | domestic | Indianapolis | IN | USA |
| 1312 | 03/10/11 | M | domestic | Oregon City | OR | USA |
| 1314 | 02/08/11 | M | domestic | Camby | IN | USA |
| 1315 | 12/31/10 | F | domestic | Indianapolis | IN | USA |
| 1316 | 03/05/11 | M | domestic | Toledo | OH | USA |
| 1317 | 12/13/10 | F | domestic | Zionsville | IN | USA |
| 1318 | 12/31/10 | F | domestic | Indianapolis | IN | USA |
| 1320 | 03/05/11 | F | domestic | Toledo | OH | USA |
| 1324 | 01/28/11 | F | domestic | Toledo | OH | USA |
| 1329 | 01/10/11 | M | domestic | Yorktown | IN | USA |
| 1331 | 02/08/11 | F | domestic | Camby | IN | USA |
| 1332 | 02/05/11 | M | domestic | Temperance | MI | USA |
| 1333 | 02/08/11 | F | domestic | Camby | IN | USA |
| 1335 | 12/31/10 | F | domestic | Indianapolis | IN | USA |
| 1336 | 03/05/11 | F | domestic | Toledo | OH | USA |
| 1338 | 03/08/11 | M | domestic | Portland | OR | USA |
| 1339 | 03/07/11 | M | domestic | Portland | OR | USA |
| 1340 | 03/03/11 | M | domestic |  | IN | USA |
| 1341 | 03/07/11 | F | domestic | Portland | OR | USA |
| 1342 | 12/31/10 | F | domestic | Indianapolis | IN | USA |
| 1343 | 03/11/11 | F | domestic | Oregon City | OR | USA |
| 1344 | 01/31/11 | M | domestic | Toledo | OH | USA |
| 1345 | 03/11/11 | M | domestic | Oregon City | OR | USA |
| 1346 | 03/07/11 | F | domestic | Portland | OR | USA |
| 1350 | 03/07/11 | M | domestic | Portland | OR | USA |
| 1351 | 03/07/11 | M | domestic | Portland | OR | USA |
| 1352 | 03/07/11 | M | domestic | Portland | OR | USA |
| 1353 | 03/07/11 | M | domestic | Portland | OR | USA |
| 1354 | 03/10/11 | M | domestic | Oregon City | OR | USA |
| 1357 | 03/03/11 | F | domestic | Vancouver | WA | USA |
| 1358 | 03/03/11 | F | domestic | Vancouver | WA | USA |
| 1360 | 03/05/11 | M | domestic | Toledo | OH | USA |
| 1366 | 04/01/11 | F | domestic | Los Angeles | CA | USA |
| 1368 | 03/10/11 | F | domestic | Oregon City | OR | USA |
| 1369 | 03/03/11 | F | domestic | Vancouver | WA | USA |
| 1371 | 03/05/11 | F | domestic | Merced | CA | USA |
| 1372 | 03/05/11 | M | domestic | Merced | CA | USA |
| 1373 | 01/31/11 | F | domestic | Ottawa Lake | MI | USA |
| 1374 | 03/07/11 | F | domestic | Portland | OR | USA |
| 1378 | 01/03/11 | F | domestic | Northwood | OH | USA |
| 1380 | 12/22/10 | M | domestic | Toledo | OH | USA |
| 1381 | 02/05/11 | M | domestic | Temperance | MI | USA |
| 1382 | 01/25/11 | M | domestic | Perrysburg | OH | USA |

**Table S2** Microsatellite multiplex PCR protocols and locus-specific details. Q-soln: QIAGEN Q solution µL; MM: master mix µL; Primers: primer mix µL; Water: molecular grade water µL; DNA: template DNA µL; Na: number of alleles; Ne: effective number of alleles; I: Shannon index; H_O_: observed heterozygosity; uH_E_: unbiased expected heterozygosity.

| Plex solution | | Reference | Q-soln | PCR MM | Primers | Water | DNA |
| --- | --- | --- | --- | --- | --- | --- | --- |
|  | Primer |  | Na | Ne | I | H_O_ | uH_E_ |
| Singleplex 1^a^ | |  | 0.00 | 6.25 | 1.25 | 3.00 | 2.00 |
|  | Lut818 | Dallas & Piertney 1998 | 7 | 1.27 | 0.50 | 0.19 | 0.21 |
| Singleplex 2^a^ | |  | 0.00 | 6.25 | 1.25 | 3.00 | 2.00 |
|  | Mvi114 | O'Connel et al., 1996 | 3 | 1.99 | 0.70 | 0.38 | 0.50 |
| Multiplex 1^b^ | |  | 1.25 | 6.25 | 1.25 | 1.75 | 2.00 |
|  | G1A | Paetkau & Strobeck, 1994 | 8 | 1.35 | 0.46 | 0.22 | 0.26 |
|  | MpuA212w | Ernest et al., 2012 | 3 | 1.38 | 0.46 | 0.22 | 0.28 |
|  | MpuA4w | Ernest et al., 2012 | 9 | 2.37 | 1.04 | 0.45 | 0.58 |
| Multiplex 2^c^ | |  | 0.00 | 6.25 | 1.25 | 3.00 | 2.00 |
|  | MpuD209w | Ernest et al., 2012 | 3 | 1.71 | 0.74 | 0.38 | 0.41 |
|  | MpuB6w | Ernest et al., 2012 | 8 | 1.75 | 0.84 | 0.36 | 0.43 |
|  | MpuD231w2 | Ernest et al., 2012 | 5 | 1.90 | 0.81 | 0.43 | 0.47 |
| Multiplex 3^b^ | |  | 1.25 | 6.25 | 1.25 | 1.75 | 2.00 |
|  | MpuA223w | Ernest et al., 2012 | 9 | 2.23 | 1.13 | 0.48 | 0.55 |
|  | MpuB12w2 | Ernest et al., 2012 | 4 | 1.75 | 0.70 | 0.36 | 0.43 |
|  | MpuB202w | Ernest et al., 2012 | 3 | 1.92 | 0.68 | 0.37 | 0.48 |
|  | MpuB217w | Ernest et al., 2012 | 7 | 2.05 | 0.83 | 0.37 | 0.51 |
|  | MpuC102w | Ernest et al., 2012 | 2 | 1.06 | 0.14 | 0.06 | 0.06 |
| Multiplex 4^b^ | |  | 1.25 | 6.25 | 1.25 | 1.75 | 2.00 |
|  | MpuA10w | Ernest et al., 2012 | 4 | 2.05 | 0.75 | 0.40 | 0.51 |
|  | MpuA229w | Ernest et al., 2012 | 5 | 1.54 | 0.68 | 0.30 | 0.35 |
|  | MpuB1w | Ernest et al., 2012 | 7 | 2.10 | 0.97 | 0.39 | 0.52 |
|  | MpuD209w | Ernest et al., 2012 | 5 | 2.57 | 1.05 | 0.49 | 0.61 |
|  | Mvi87 | O'Connel et al., 1996 | 5 | 1.42 | 0.63 | 0.12 | 0.29 |
| Multiplex 5^c^ | |  | 0.00 | 6.25 | 1.25 | 3.00 | 2.00 |
|  | MpuB112w2 | Ernest et al., 2012 | 6 | 2.40 | 0.98 | 0.50 | 0.58 |
|  | MpuD207w | Ernest et al., 2012 | 4 | 2.24 | 0.94 | 0.48 | 0.55 |
|  | EluD311w | Lam et al., 2016 | 4 | 1.11 | 0.24 | 0.09 | 0.10 |
|  | Lut435 | Dallas & Piertney, 1998 | 7 | 1.80 | 0.78 | 0.36 | 0.44 |
|  | Lut604 | Dallas & Piertney, 1998 | 8 | 1.56 | 0.77 | 0.23 | 0.36 |
| Multiplex 6^d^ | |  | 1.25 | 6.25 | 1.25 | 1.75 | 2.00 |
|  | MpuA231w | Ernest et al., 2012 | 8 | 2.26 | 1.01 | 0.48 | 0.56 |
|  | Mel10 | Domingo-Roura et al., 2003 | 8 | 1.85 | 0.72 | 0.38 | 0.46 |
|  | Mvi111 | O'Connel et al., 1996 | 7 | 2.38 | 1.02 | 0.46 | 0.58 |
| Multiplex 7^b^ | |  | 1.25 | 6.25 | 1.25 | 1.75 | 2.00 |
|  | MpuA115w | Ernest et al., 2012 | 7 | 1.65 | 0.75 | 0.34 | 0.39 |
|  | MpuA121w | Ernest et al., 2012 | 5 | 1.77 | 0.72 | 0.36 | 0.43 |
|  | MpuA129w | Ernest et al., 2012 | 4 | 1.25 | 0.39 | 0.17 | 0.20 |
|  | MpuB9w | Ernest et al., 2012 | 6 | 1.76 | 0.89 | 0.37 | 0.43 |
|  | MpuC4w2 | Ernest et al., 2012 | 5 | 1.53 | 0.71 | 0.29 | 0.35 |
| ^a^15 min 95C; 35 cycles of 94C for 30sec, 55C for 90sec, 72C for 90sec; 60C for 30min | | | | | | | |
| ^b^15 min 95C; 40 cycles of 94C for 30sec, 60C for 90sec, 72C for 90sec; 72C for 10min | | | | | | | |
| ^c^15 min 95C; 35 cycles of 94C for 30sec, 60C for 90sec, 72C for 90sec; 60C for 30min | | | | | | | |
| ^d^15 min 95C; 40 cycles of 94C for 30sec, 58C for 90sec, 72C for 90sec; 72C for 10min | | | | | | | |
